# Recent Advances in Metal-Oxide-Based Photoresists for EUV Lithography

**DOI:** 10.3390/mi15091122

**Published:** 2024-08-31

**Authors:** Muhammad Waleed Hasan, Laura Deeb, Sergei Kumaniaev, Chenglu Wei, Kaiying Wang

**Affiliations:** Department of Microsystems, University of South–Eastern Norway, 3184 Horten, Norway; waleed.hasan.2502@gmail.com (M.W.H.); lauradeeb2631999@gmail.com (L.D.); sergei.kumaniaev@outlook.com (S.K.); milu0229@163.com (C.W.)

**Keywords:** inorganic photoresist, Zr, Hf, Zn, tin (Sn) oxides, EUV lithography, high-resolution EUV, 3D printing, photon absorption, CMOS

## Abstract

Extreme ultraviolet lithography (EUVL) is a leading technology in semiconductor manufacturing, enabling the creation of high-resolution patterns essential for advanced microelectronics. This review highlights recent progress in inorganic metal-oxide-based photoresists, with a focus on their applications in EUVL. The unique properties of zinc-based, tin–oxygen, and IVB group inorganic photoresists are examined, showcasing their enhanced chemical reactivity and precise patterning capabilities. Key advancements include the development of zinc oxide and tin oxide nanoparticles, which demonstrate significant improvements in photon absorption and solubility under extreme ultraviolet exposure. Additionally, the review delves into the photochemical reactions of tin–oxygen clusters and the influence of various ligands on film density and cross-linking. The findings suggest that these inorganic photoresists not only improve photolithographic performance but also hold potential for broader applications, such as pyroelectric infrared sensors and 3D printing. Future research directions are outlined, including the optimization of process parameters, the exploration of new ligand and metal combinations, and the evaluation of the environmental benefits of inorganic photoresists over traditional organic ones. These advancements are poised to further enhance the resolution and patterning capabilities required for next-generation semiconductor devices.

## 1. Introduction

At the end of the 20th century, EUVL emerged as a promising technology for high-resolution patterning in semiconductor manufacturing [1]. The escalated demand for powerful and energy-efficient microchips has given EUVL a powerful development impetus. Since 2019, following two decades of maturation, EUVL has become the leading lithography technique for high-volume manufacturing (HVM), replacing traditional deep ultraviolet lithography (DUVL) and its predecessor, visible light lithography (VLL) [2,3].

**Figure 1:**
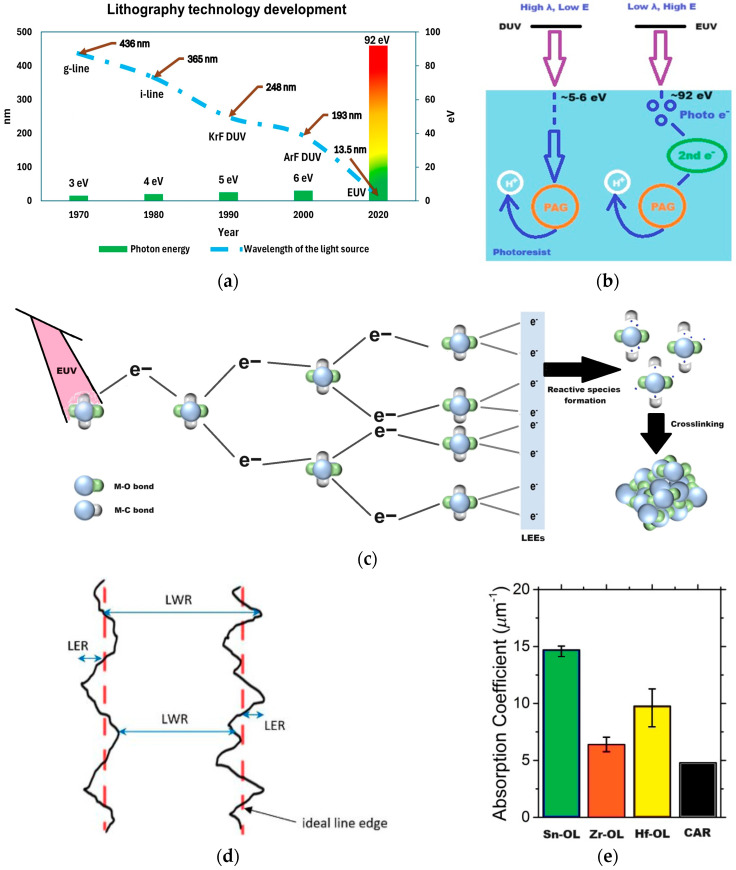
(**a**) Lithography technology advancement from 1970 to 2020. (**b**) DUV and EUV lithography comparison [6]. (**c**) Photochemical reactions of EUV photoresist (reprinted with permission from ref. [5], 2023, Elsevier). (**d**) Definitions of line edge roughness and line width roughness (reprinted with permission from ref. [7], 2016, Elsevier). (**e**) Absorption coefficient of different metal oxide photoresists at EUV as compared to traditional CAR (reprinted with permission from ref. [8], 2002, SPIE).

According to Rayleigh’s criterion, shorter wavelength light sources enable the creation of nanostructures with smaller feature sizes [4]. At the dawn of lithography technology development, the g-line of a Hg lamp with a wavelength of 436 nm has been used as an exposure tool, later followed by the i-line with a 365 nm wavelength. Subsequently, DUVL employed wavelengths ranging from 248 nm with KrF lasers to 193 nm with ArF lasers [5]. The latter technology became the primary method for high-volume manufacturing (HVM) starting in the late 1990s. It remained dominant until it was surpassed by EUV lithography, which utilizes a light source with a wavelength of only 13.5 nm. The historical timeline of these lithography standard enhancements is represented in Figure 1a.

However, significant progress in lithography techniques is accompanied by certain challenges. A reduction in light source wavelength increases photon energy, leading to pronounced photoresist degradation and stochastic effects, which can increase line edge roughness (LER) and line width roughness (LWR) of the features [9].

In contrast, the photon energies associated with DUVL are insufficient to excite the electrons within the molecules of the photoresist. As a result, after the exposure of a photoresist to DUV light, the photoacid generator (PAG) within the resist absorbs the photons, and the consumed energy excites the PAG molecules. The further chemical reactions cause deprotection of polymer chains in the resist, which alter the solubility of the resist. In EUVL, these processes take place only after non-chemical interactions, as illustrated in Figure 1b [6].

With the technology advancement, the transition to EUV lithography has significantly increased photon energy, up to 92 eV [10]. This rise in photon energy changed the chemistry behind lithography processes, offering both advantages and new challenges.

On the one hand, both valence and internal valence electrons can be excited by high-energy EUV photons, producing photo-emitted electrons that initiate reactions in the surrounding material, generating secondary electrons [11]. These secondary electrons have enough energy to excite additional atoms, creating a cascade of electron interactions [10]. The energy from the electrons is dissipated through multiple inelastic collisions, resulting in a broad distribution of lower-energy secondary electrons (LEEs) capable of inducing further chemical changes within the resist [12]. Finally, the ionization and excitation of resist molecules produce highly reactive species, which initiate chemical reactions, such as cross-linking (Figure 1c) and chain scission. These processes contribute to the overall enhanced resolution of lithography and enable the fabrication of much finer features on the wafer [5].

Conversely, higher photon energy in EUV indicates that the energy dose of EUV radiation consists of fewer photons compared to the same energy dose of DUV radiation. Therefore, it becomes more challenging to activate chemical reactions in the photoresist that are crucial to change its solubility [11]. High EUV doses pose significant issues for traditional chemically amplified photoresists (CARs), slowing manufacturing throughput [13]. Moreover, current EUVL photoresist systems require significant optimization for ultrahigh-resolution patterning. Thus, the microfabrication industry is in high demand for cutting-edge photoresists capable of efficiently absorbing EUV photons. Consequently, researchers are currently focusing on developing new-generation photoresists with enhanced efficiency.

Photoresist material selection depends on balancing two factors: high transmissivity, ensuring uniform exposure and minimizing LER and LWR (Figure 1d), and high EUV light absorption, maximizing the use of incoming radiation [8].

Research by Fallica et al. [8] confirmed that metal oxides absorb EUV light more efficiently than organic polymers. Tin–oxide photoresists demonstrated the highest absorption rates, 2 to 3 times greater than polymer-based photoresists. Hafnium oxide and zirconium oxide photoresists also showed superior absorptivity in the EUV range [8]. These results are presented graphically in Figure 1e.

Moreover, metal-oxide-based photoresists possess high etch resistivity meaning that they can withstand the etching processes used to transfer patterns into underlying layers more effectively [9]. In addition, a study [14] shows that metal-oxide-based photoresists, especially tin-oxide-based ones, have enhanced sensitivity because of the higher density of reactive species that are formed as the result of interactions with EUV photons. Furthermore, upon exposure metal oxide resists form a highly cross-linked inorganic network, which provides superior mechanical and thermal stability and, therefore, helps to maintain pattern fidelity [15]. Photoresists based on zirconium and hafnium oxo (Zr oxo and Hf oxo) have also shown impressive EUV patterning capabilities and demonstrated ability to perform well at extremely fast photo speeds, which makes them well-suited for advanced manufacturing processes [16].

Metal-oxide-based photoresists have also demonstrated high potential in industry applications. For instance, Inpria Corporation achieved a resolution of 8 nm half-pitch and high etch selectivity using hafnium-based photoresists, although initial issues with low EUV photosensitivity were later resolved with tin-oxide-based resists, reaching a 13 nm half-pitch [17].

This paper explores the pioneering advancements of metal-oxide-based photoresists in EUV lithography, highlighting the performance of zinc-based, tin oxide, and IVB group inorganic photoresists. Through an examination of specific properties, recent advances, characterization techniques, performance evaluation, and industry applications, this study reveals their potential to meet the stringent resolution and sensitivity requirements in the ever-evolving semiconductor manufacturing industry.

## 2. Metal-Oxide-Based Photoresist Chemistry

Zinc-based inorganic photoresists, incorporating zinc oxide as the photoactive component, offer enhanced chemical reactivity upon light exposure, crucial for high-resolution patterning. Binders, namely metal oxides or organic polymers, optimize EUV photon absorption, with carboxylate group ligands playing a key role. Tin–oxygen photoresists, composed of tin oxo clusters, exhibit negative-tone properties, demonstrating effectiveness in EUV lithography. IVB group metal oxides, including hafnium and zirconium, enhance etch resistance and resolution through reduced organic content and careful ligand selection. These advancements in inorganic photoresist mechanisms underscore their potential in achieving precise and efficient semiconductor manufacturing [18].

### 2.1. Zinc-Based Inorganic Photoresist Mechanism

Zinc-based inorganic photoresists have zinc oxide (ZnO) as the photoactive component, which ensures that the photoresist will have the ability to be chemically reactive when exposed to light. For the mechanical stability and the control of the adhesion and film-forming properties of the photoresist, binders or matrix materials are used, e.g., metal oxides, such as alumina or silica, or organic polymer, such as polyacrylic acid.

Thakur et al. [19] used carboxylate group ligands (e.g., methacrylate), which are also considered organic polymers, and their usage played a key role in achieving the highest EUV photon absorption levels. Similar to other types of inorganic photoresists, zinc-based photoresists contain solvents and additives, which help to dissolve the components of the photoresist formulation and adjust its viscosity for coating or application onto substrates and improve the performance or processing characteristics of the photoresist, correspondingly. Some ZnO-based photoresists may contain a photoinitiator which is utilized to enhance the photochemical reaction upon exposure to light. When the photoresist undergoes the light treatment, the ZnO nanoparticles absorb photons of light energy, after which, as the result of photochemical reactions, reactive species are generated. They could include free radicals, electrons, or holes. These reactive species also lead to another chemical reactions, which contribute to changes within the chemical structure of the material, such as polymerization, cross-linking, or degradation.

**Figure 2:**
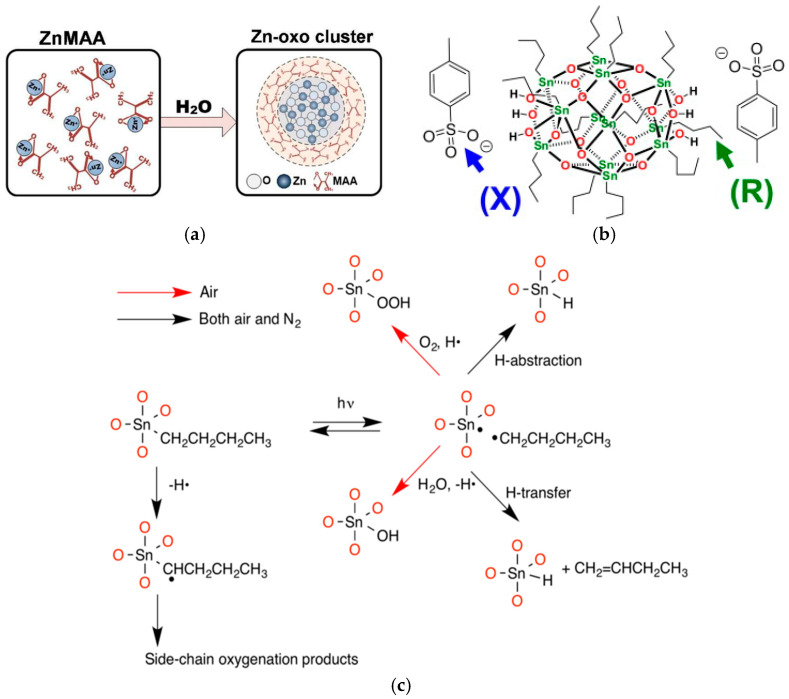
(**a**) Hydrolysis–dehydration chemical reaction for Zn oxo cluster formation (reprinted with permission from ref. [23], 2012, Royal Society of Chemistry). (**b**) Structure of tin oxo clusters (reprinted with permission from ref. [21], 2014, Elsevier). (**c**) Photochemical reactions for TinOH subjected to UV in air and $N_{2}$ [22].

One of the most critical factors affecting the performance of photoresists is the extent of EUV light absorption [19]. For zinc-based photoresists, which have metal oxo clusters as molecular compounds, the dense Zn metallic oxo core is surrounded by trifluoroacetate and methacrylate ligands. These ligands enhance solubility and contribute to increased EUV photon absorption due to the presence of fluorine [20]. The formation of zinc oxide clusters occurs through a hydrolysis–dehydration chemical reaction (Figure 2a).

Therefore, zinc-based inorganic photoresists incorporate ZnO as the photoactive component and offer enhanced chemical reactivity upon exposure to light, crucial for achieving high-resolution patterning. Additionally, the use of binders or matrix materials such as metal oxides or organic polymers, exemplified by carboxylate group ligands, plays a pivotal role in optimizing EUV photon absorption levels. Furthermore, in photolithography applications, zinc-based inorganic photoresists are coated onto substrates, exposed to light through a photomask, and undergo selective removal of exposed regions during development, highlighting their potential for precise patterning in semiconductor manufacturing.

### 2.2. Tin–Oxygen Inorganic Photoresist Mechanism

Tin–oxygen inorganic photoresists are typically composed of tin oxide (${SnO}_{2}$) nanoparticles dispersed in a solution. These particles are arranged in a specific structure consisting of tin and oxygen atoms, which are called tin oxo clusters. The $\left[{\left(RSn\right)}_{12}O_{14}{\left(OH\right)}_{6}\right]X_{2}$ tin oxo clusters (Figure 2b) comprise tin oxo cages in a spherical shape, where covalent bonds bind each of the twelve tin atoms to a single organic (R) group [21]. The clusters carry a bivalent charge and are typically associated with anion pairs (X−).

The experiments by Cardineau et al. [21] on photolithographic properties of tin oxo clusters revealed their photochemical mechanism. The study showed that the working principle of tin oxide photoresists involves the photodecomposition and subsequent cross-linking reactions initiated by EUV exposure. The primary reaction mechanism is homolytic cleavage of the tin–carbon (Sn–C) bond, which produces tin-centered radicals. These radicals initiate reactions with adjacent tin oxo clusters, leading to the formation of cross-links and ultimately the agglomeration of clusters, which is essential for the resist’s function. In studying the photodecomposition mechanism, various tin oxo clusters were prepared with different carboxylate anions, which have bond dissociation energies ranging from 67 to 103 kcal/mol. The research found that photoreaction occurs with the tin oxide cation rather than the counterions, which act merely as non-reactive spacers inhibiting the clusters from combining. Another potential reaction during EUV exposure is the homolytic cleavage of the relatively weak Sn–C bond (~50 kcal/mol), which produces stable tin-centered radicals. This hypothesis was tested by synthesizing a series of tin clusters with alkyl groups of varying radical stabilities and evaluating their sensitivity in printing 50 nm dense lines. The results showed that resist sensitivity correlated more closely with the Sn–C bond strength than with the carbon–carboxylate bond strength, with clusters having weaker Sn–C bonds exhibiting better sensitivity. This suggests that homolytic cleavage of the Sn–C bond is crucial, as it produces tin-centered radicals that facilitate cross-linking. Moreover, large counterions increase the space between tin oxo clusters, thereby reducing the likelihood of tin–tin bond formation and decreasing the resist sensitivity. This indicates that the spatial arrangement of the clusters, influenced by the size of the carboxylate anions, also plays an important role in the photoresist’s performance [21].

The group of Zhang et al. [22] have also studied the photochemical reactions occurring in tin oxo cage clusters. The photoresist film was prepared by spin-coating a filtered tin oxo solution onto conductive Cr/Au-coated glass, followed by baking, ultraviolet (UV) exposure, and characterization using hard X-ray photoelectron spectroscopy to identify photochemical reaction pathways (Figure 2c). The lowest electronically excited triplet state has been found to be of the σ → d type, where the Sn–C bond is readily split into two radicals. The Sn radicals can react directly with oxygen and water, increasing the oxidation states of Sn atoms. The butyl radical can donate a hydrogen atom or abstract one from neighboring cages to form volatile stable molecules like butane or butene, which can easily escape into a vacuum. A hydrogen atom may also be abstracted from a butyl group, forming a C-centered radical that can further react to produce side chain oxygenation products. For samples exposed in $N_{2}$, the chance for radicals to recombine is greater, resulting in less C loss compared to samples exposed in air. In the cage, cleaved butyl radicals can also react with O to generate a C-O bond. Another potential reaction is the formation of butane and a tin hydride when a hydrogen atom is transferred from the butyl radical to the Sn radical. The reason why post-exposure baking enhances the development of insoluble material after exposure to EUV could be attributed to the formation of oxidation-sensitive but otherwise stable reaction products.

Therefore, by investigation of the features and chemistry behind the tin-oxo-based photoresists, it has been confirmed in practice that in EUV lithography they can be effectively used as a negative photoresist.

### 2.3. IVB Group Inorganic Photoresist Mechanism

Hafnium oxide photoresists and zirconium oxide photoresists (with Zr-MAA ligands) have shown significant potential in enhancing etch resistance and achieving high-resolution pattern transfer. Research by Trikeriotis et al. [24] demonstrates that reducing the organic content in hafnium oxide photoresists can improve these characteristics by 2–3 times. Similarly, zirconium oxide has proven effective as a primary material for inorganic photoresists [25].

Toriumi et al. investigated photoresists made of metal oxides and organic molecules, focusing on composites of zirconium oxide (ZrOx), titanium oxide (TiOx), and methacrylic acid (MAA) ligands [26]. Scanning transmission electron microscopy (STEM) was used to characterize the particle morphology and X-ray photoelectron spectroscopy (XPS) to analyze surface composition before and after EUV exposure [26]. The STEM results showed that the ZrOx core in ZrOx-MAA had better dispersibility compared to the TiOx core in TiOx-MAA, which tended to agglomerate. This difference was attributed to the stronger interaction between the ZrOx core and shell molecules, whereas MAA and TiOx had stronger interactions with themselves. XPS analysis revealed that the ZrOx-MAA photoresist experienced partial decomposition of surface ligand molecules after exposure, leading to an increase in the electron density of Zr atoms. Similarly, Trikerioti et al. used FTIR and XPS to verify that Zr-MAA lost some carboxyl ligands after EUV exposure [24].

**Figure 3:**
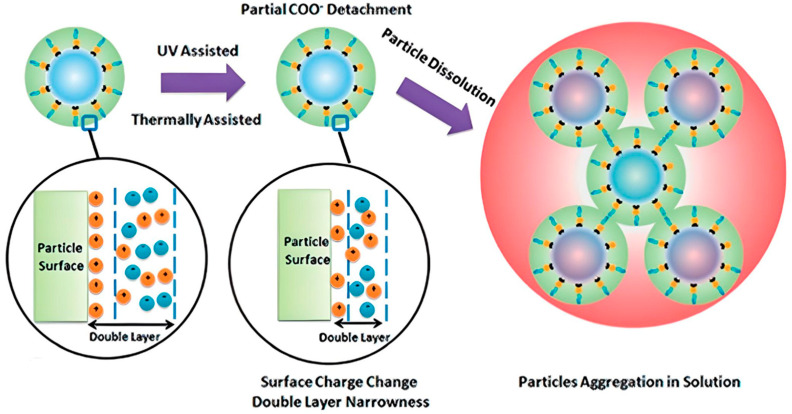
Increase in size of HfO_2_ particle mechanism after UV treatment [27].

By examining the properties of particles before and after illumination using thermogravimetric analysis (TGA), XPS, and dynamic light scattering (DLS), it was found that UV illumination led to a minor dissociation of ligands from the surface of metal particles. This dissociation altered the surface charge of metal oxide nanoparticles, causing aggregation, an increase in particle size, and changes in solubility (Figure 3). The study revealed that $HfO_{2}$ modified with MAA and DMA ligands was more prone to agglomeration under UV light compared to $HfO_{2}$ modified with benzoic acid (BA) ligands. This difference was attributed to the stronger interaction between BA and the metal oxide particles, resulting in a more stable formation of metal oxide particles/ligands [27].

In a study [18], grazing incidence X-ray scattering (GIXS) was utilized to analyze the photoresist film, revealing that the particles were disordered and showed minimal aggregation. This indicates that the metal oxide photoresist does not require significant ligand loss or extensive polymerization to alter its solubility after exposure. Instead, the solubility changes may be attributed to other factors, such as the coupling of terminal C-C double bonds.

### 2.4. Absorptivity Comparison under EUV

At EUV wavelengths (13.5 nm), the exposure photochemistry of resists significantly differs from that of previous technologies. Interaction between EUV light and the electronic states of elements is considerably stronger than the interaction between DUV light and molecular materials, leading to a higher absorption of EUV light by matter. Achieving higher lithographic resolution requires the use of thinner coatings (approximately 35 nm) for EUV compared to DUV to prevent pattern collapse. However, an organic photoresist film of this thickness, with a typical absorption of 4.8 $\mu m^{-1}$, would only utilize about 15% of the incident light. This is a critical consideration for high-volume manufacturing, as generating EUV light is expensive, and EUV sources, typically based on tin plasma, have low efficiency (around 5%) and limited collector lifetimes [28].

**Figure 4:**
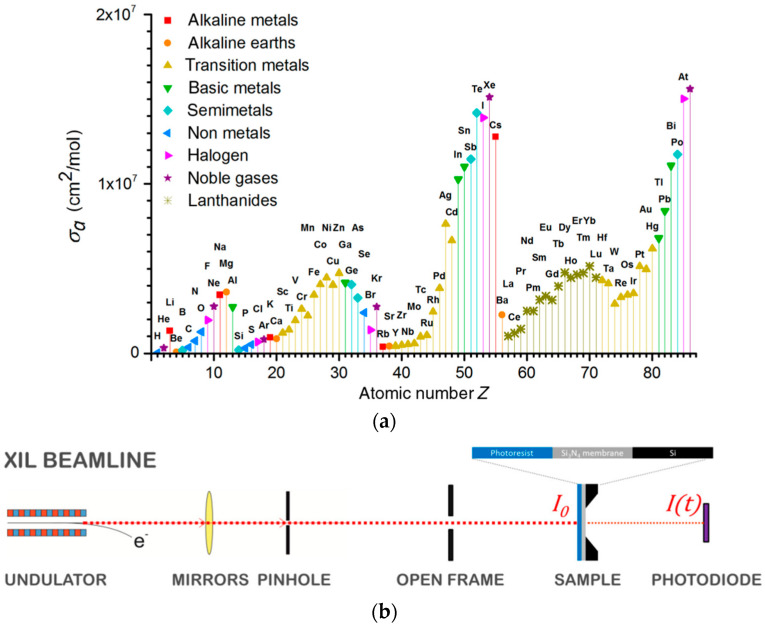
(**a**) Atomic absorption of different elements at EUV; (**b**) Experimental setup for absorption measurement (reprinted with permission from ref. [8], 2002, SPIE).

The absorption of EUV light in matter is entirely dependent on the atomic composition and the absorption cross-section of the elements involved. In Figure 4a, based on tabulated data, there are significant differences in absorption cross-sections among elements. Transition metals, along with metals and semimetals with high atomic numbers, exhibit deep semicore orbitals with large interaction cross-sections with EUV photons, making them promising candidates for enhancing optical absorption in photoresists.

There have been a few experimental studies measuring the absorption coefficients of photoresist materials in the EUV range. One notable study discovered a correlation between the transmissivity of photoresists at EUV wavelengths and the amount of incorporated tellurium [29]. Measuring absorption in thin films directly poses several challenges. Because EUV light is strongly absorbed by matter, achieving measurable signals in transmission mode requires a bright and stable source, which is more difficult to obtain at EUV wavelengths compared to longer ones. Furthermore, absorption measurements should be performed on the photoresist in its coated form to replicate actual exposure conditions. This necessitates the use of a transparent substrate for EUV, such as a free-standing membrane thinner than a micron. Accurately measuring the thickness of the spin-coated film is also crucial for determining the absorption coefficient.

A technique for evaluating thin layer photoresist absorption under EUV light from a synchrotron source in transmission mode has been developed by Fallica et al. [8]. The experimental setup allows determination of the absorption coefficient by measuring the transmissivity and thickness of the thin photoresist films, accounting for all sources of uncertainty. In this study, they examined the absorption coefficients of non-proprietary metal-based photoresists, specifically those based on tin cage structures and zirconium- and hafnium oxo clusters. The photoresist sample acquired from the Advanced Research Center for Nanolithography (ARCNL) were coated with a thickness lying in the range of 11 to 76 nm and exposed under a dose of <20 mJ/cm^2^ which is a requirement for high industrial yield. Detailed information on the composition and density of these materials enabled them to accurately calculate the expected absorption and compare it with experimental results.

The XIL-II beamline at the Swiss Light Source functions at an EUV wavelength of 13.5 nm, employing a synchrotron source that delivers an average flux exceeding 30 mW/cm^2^. The beamline is primarily used for EUV interference lithography and actinic mask inspection, and the beam is produced by an undulator source, which is focused through a pinhole with a 30 µm diameter. Subsequently, the beam expands over approximately 12 m, resulting in a relatively uniform beam profile across several millimeters. A 0.5 × 0.5 mm^2^ open-frame mask is placed in front of the sample for these operations.

The AXUV100G silicon photodiode is placed behind the sample in the experimental setup, which was created in a previous Fallica study [8]. Photocurrent is measured using a Keithley 6430 ammeter. The transmittance of a blank SiNx membrane is used to calibrate the net flux (I_0_), as the membrane itself absorbs EUV light. The ratio of the measured photocurrent (I) to the reference photocurrent yields the transmittance (T_X_) of the thin photoresist layer. The experimental setup is depicted in Figure 4b.

The absorption coefficients (α) of metal-containing resists were experimentally measured and compared with a standard organic CAR having a coefficient of 4.8 µm^−1^. As shown in Figure 1e, tin-based compounds exhibited absorption coefficients that were two to three times greater than those of conventional organic CARs. Zirconium-containing resists showed only a minor increase in absorption, which is attributed to zirconium’s relatively small absorption cross-section compared to oxygen and carbon. Conversely, the hafnium-based material (HfM) displayed a significantly higher absorption coefficient of about 9.0 µm^−1^, due to hafnium’s superior absorptivity compared to conventional organic resists.

## 3. Metal-Oxide-Based Photoresist Synthesis Techniques

### 3.1. Novel Synthesis Techniques for Metal Oxides and EUV Lithography Applications

#### 3.1.1. Atomic Layer Deposition (ALD)

ALD is a pivotal technology in semiconductor fabrication, enabling the precise deposition of thin films crucial for sub-5 nm node devices in EUV lithography. By alternating exposures to inorganic and organic precursors, ALD achieves uniform, atomically precise coatings, overcoming the limitations of traditional photoresists. This method enhances EUV absorption, mechanical strength, and etch resistance, which are critical for maintaining pattern fidelity at ultrafine scales [30]. The following sections discuss ALD-based techniques and their roles.

**Figure 5:**
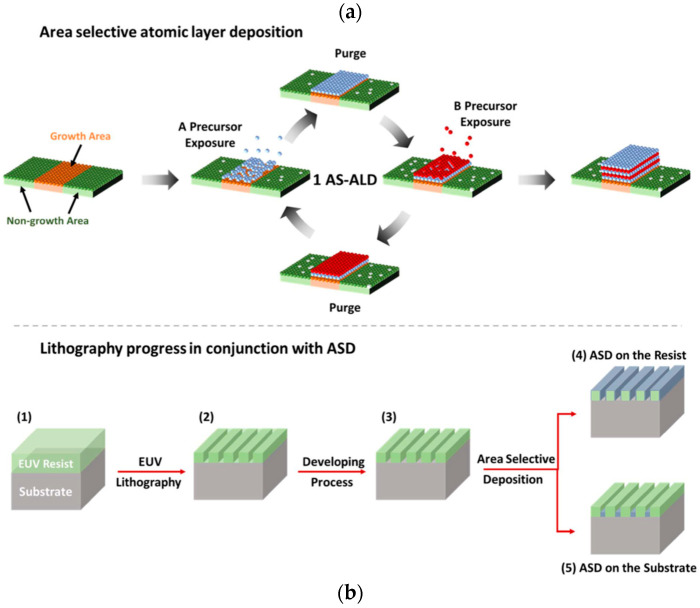
(**a**) Diagram showing the area-specific ALD process. (**b**) The EUV patterning process flow in combination with the area-selective deposition method: (1) EUV resist spin-coating on substrate, (2) exposure of the pattern via EUV lithography, (3) development and materials applied to (4) patterned EUV resist or (5) substrate on a selective basis [30].

- Area-Selective Deposition (ASD) atomic layer deposition and its derivatives for extreme ultraviolet (EUV) photoresist applications
  o ASD is a strategic additive method utilized within the semiconductor industry to enhance device performance through precise material deposition. This technique, implementable via ALD or chemical vapor deposition (CVD), allows for the targeted growth of thin films on specific regions of a substrate, supporting applications such as selective epitaxial growth [31] and cobalt capping of copper [32] (Figure 5a). Recent advancements have expanded ASD’s application to post-development steps in EUV lithography, where selective deposition on EUV patterned resists significantly reduces defect densities and improves pattern fidelity [33,34] (Figure 5b).

ASD’s capability to precisely target damaged sidewalls of EUV patterns directly addresses the challenges of LER and LWR. Studies, such as those by Wada et al., have demonstrated reductions in LWR and LER by approximately 26% and 35%, respectively [33]. Furthermore, Liu et al. showed that ASD integration with EUV self-aligned double patterning leads to a progressive decrease in LER as feature critical dimension (CD) reduces [35].

The application of ASD as a post-lithography treatment has also been pivotal in reducing defect densities, especially in thinner resists. For instance, Lutker-Lee et al. reported a significant reduction in defect occurrences, such as line breaks, equating defect densities in thinner resists to those in standard thicknesses. The infusion of more etch-resistant materials like ${TiO}_{2}$, selectively deposited on polymers such as poly(tert-butyl methacrylate) (PtBuMA), not only aids in hardening the organic resists but also facilitates the construction of higher aspect ratio features crucial for advancing EUV lithography capabilities [30].

- Vapor-Phase Infiltration (VPI)

**Figure 6:**
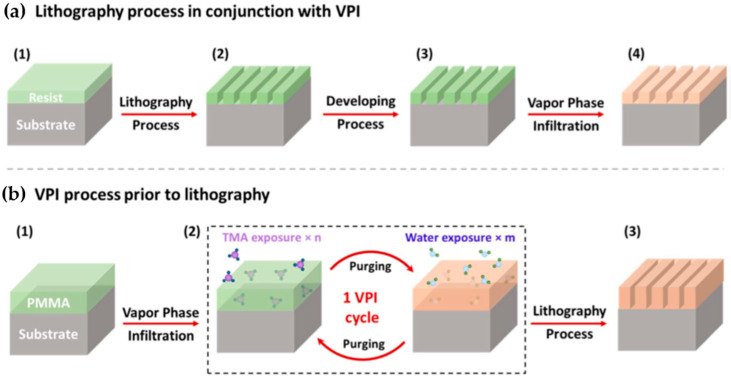
(**a**) Workflow for patterning integrated with VPI: (1) applying resist onto a Si substrate using spin-coating, (2) exposing the pattern using e-beam or EUV lithography, (3) development step, and (4) SIS $AlO_{x}$ infiltration. (**b**) Workflow for VPI before lithography: (1) applying resist onto the substrate using spin-coating, (2) infiltrating with $AlO_{x}$, and (3) pattern exposure using e-beam or EUV lithography [30].

VPI, derived from ALD, is an emerging method that hybridizes polymer-based materials by embedding inorganic elements into an organic matrix. This technique distinguishes itself from ALD through the infiltration of gaseous metal precursors into polymers during the exposure step, enhancing the properties of EUV photoresists by forming inorganic–organic composites [36]. It differs from ASD as an inorganic precursor is used to infuse the patterned resist leading to an inorganic–organic composite formation (Figure 6a). A variety of infiltration protocols such as sequential infiltration synthesis (SIS) and sequential vapor infiltration (SVI) allow tailored improvements in LER and material robustness. For instance, the SIS process has successfully reduced LER in EUV polymer-based resists by introducing trimethylaluminum (TMA) and water to form aluminum oxide ($AlO_{x}$) networks within the resist structure, improving etch resistance and structural integrity [37].

The practical applications of VPI in post-lithography treatments have shown substantial benefits in pattern performance and defect reduction. Notably, LER reductions and the robustness of etch masks have been significantly enhanced, allowing for the fabrication of higher aspect ratio features with improved depth control during etching processes. Moreover, VPI has been applied ex situ to pre-lithography coatings (Figure 6b) to generate hybrid inorganic–organic resist materials, significantly increasing EUV sensitivity and mechanical strength of the resists. It also includes innovative applications such as poly methyl methacrylate (PMMA) infiltrated with $AlO_{x}$ as a positive-tone hybrid EUV resist system, which demonstrated increased etch resistance and process stability through a higher level of cross-linking, crucial for advanced patterning techniques [38]. This method’s ability to adapt and modify resist properties pre- and post-lithography highlights its versatility and potential in addressing the evolving needs of EUVL technologies.

- Molecular atomic layer deposition (MALD)

**Figure 7:**
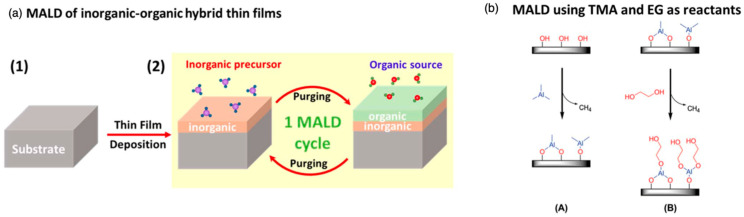
(**a**) The procedure of the MALD method, which involves the growth of hybrid thin films by sequentially exposing inorganic and organic precursors. (**b**) The surface reactions in the MALD formation of poly(aluminum ethylene glycol) alucone, utilizing TMA (A) and EG (B) as the inorganic and organic precursors, respectively [30].

MALD is an innovative extension of ALD that enables the deposition of highly conformal, organometallic thin films directly onto substrates without the need for spin-coating. This technique offers atomic-scale precision in film thickness and uniformity, making it ideal for the fabrication of EUV photoresists. Unlike traditional ALD, MALD utilizes organic moieties along with ALD precursors to grow organic-containing films, facilitating the development of hybrid inorganic–organic structures (Figure 7) suitable for advanced applications like flexible displays and photovoltaic devices [30].

The potential of MALD for creating novel, dry EUV resists has been explored. Studies have demonstrated that MALD can be effectively used to deposit and develop resists entirely in the vapor phase, addressing some of the challenges associated with conventional photoresist applications. For instance, Shi et al. used MALD to prepare Hf-based hybrid thin films that exhibit negative-tone resist characteristics due to their selective dissolution behavior under electron beam exposure [39]. This study demonstrated the ability to achieve high-resolution patterning with critical exposure doses significantly lower than in traditional methods. Additionally, the organic components in the MALD films undergo specific reactions upon electron beam irradiation, leading to changes in material properties that are crucial for EUV lithography.

Further research by Le et al. explored MALD films composed of TMA and hydroquinone (HQ), demonstrating the utility of the technique in generating negative-tone patterns using low-energy electron exposure [40]. These studies also employed in situ Fourier-transform IR (FTIR) spectroscopy to elucidate the chemical transformations within the films, providing insights into the mechanisms of solubility switching and cross-linking induced by electron interactions. These advancements underline MALD’s capability to innovate within the realm of EUV resist technology, offering pathways to enhance resist performance through molecular-level control and customization.

#### 3.1.2. Non-Aqueous Sol–Gel

Non-aqueous sol–gel techniques represent a critical advancement in the synthesis of metal oxide nanoparticles, especially significant for applications in EUV lithography. This method utilizes organic solvents instead of water and enables precise control over particle size, shape, and crystal structure at moderate temperatures, addressing many challenges associated with traditional aqueous sol–gel processes. By avoiding water, non-aqueous sol–gel not only reduces the complexity and improves the reproducibility of nanoparticle synthesis but also enables the formation of highly crystalline and uniformly shaped nanoparticles. This approach is a successful alternative to aqueous systems in the synthesis of different metal oxide nanoparticles, including IVB group oxides [41], which are used in photoresist EUVL [18]. Additionally, it provides insights into the formation of their oxo clusters [41]. The production of metal oxides for EUV lithography, where the quality and consistency of nanoparticles can significantly impact device performance and scalability, demonstrates the growing importance of non-aqueous sol–gel techniques.

Non-aqueous sol–gel chemistry is an advanced approach for synthesizing metal oxides from organometallic precursors such as metal alkoxides and acetylacetonates, which are particularly useful in environments where water cannot be used due to the sensitivity of the reactants. This method primarily utilizes solvents that supply oxygen atoms necessary for the formation of metal oxide networks, which are critical for constructing the oxidic framework without the introduction of water [42].

**Scheme 1:**
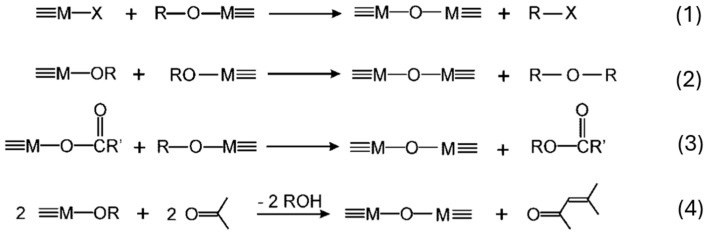
Condensation reactions forming metal–oxygen–metal bonds in non-aqueous sol–gel processes include: alkyl halide elimination (Equation (1)), ether elimination (Equation (2)), ester elimination (Equation (3)), and aldol-like condensation (Equation (4)) [42].

The transformation of these precursors into oxidic solids involves key condensation reactions that form metal–oxygen–metal bonds:

- Through condensation of metal halides and metal alkoxides, metal oxides are formed alongside the release of alkyl halides. This process is exemplified in the synthesis of anatase nanocrystals from titanium isopropoxide and titanium chloride [43] ((1) in Scheme 1).
- Ether elimination involves the condensation of two metal alkoxides, leading to the formation of a metal–oxygen–metal bond while releasing organic ether. This reaction pathway has been utilized for synthesizing hafnium oxide nanoparticles [44] ((2) in Scheme 1).
- Ester elimination takes place between metal alkoxides and carboxylates, a method applied in the synthesis of zinc oxide [45], titania [46], and indium oxide [47] ((3) in Scheme 1).
- Amide elimination facilitates the controlled growth of nanostructures like titania nanorods through the reaction of metal oleates with amines [48] ((4) in Scheme 1).

**Scheme 2:**
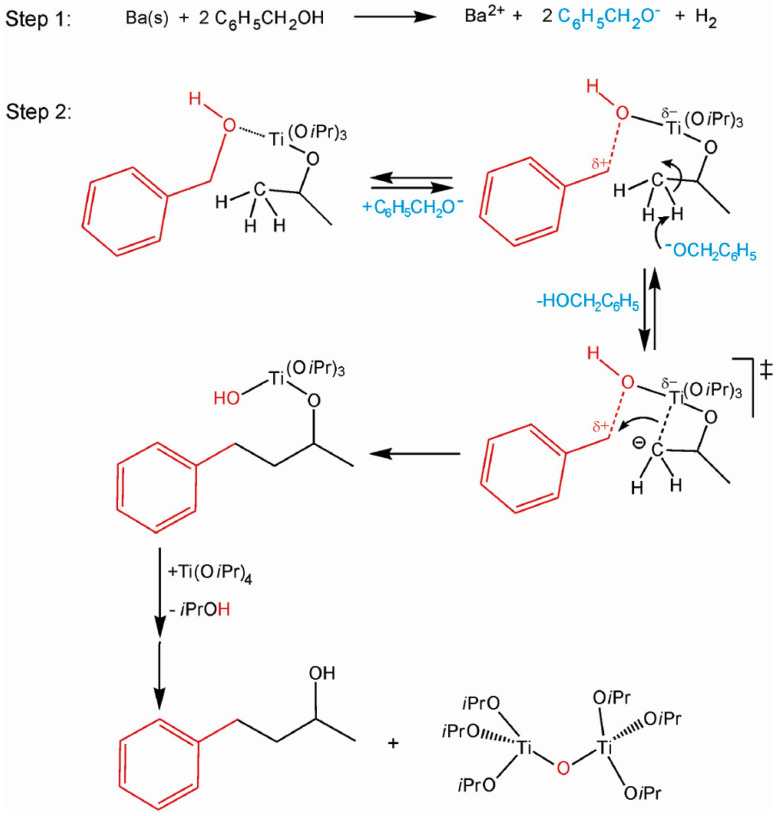
Suggested reaction pathway for creating a C–C bond, resulting in the concurrent production of $BaTiO_{3}$ nanoparticles and 4-phenyl-2-butanol [42].

An example of a more complex reaction pathway is found in the synthesis of $BaTiO_{3}$ nanoparticles in benzyl alcohol, where the C–C coupling mechanism similar to the Guerbet reaction leads to the formation of 4-phenyl-2-butanol. This reaction mechanism excludes ether elimination and instead involves the creation of a C–C bond between the isopropoxy ligand of titanium isopropoxide and benzyl alcohol. Subsequently, there is a transfer of hydroxyl groups and condensation leading to the formation of metal oxide [49] (Scheme 2).

These non-aqueous reactions underscore the crucial role of the solvent and specific conditions in directing the synthesis pathway, providing nuanced control over the chemical structure and crystallinity of the nanoparticles produced.

Non-aqueous Sol–gel Pathways for Creating Metal Oxide Nanoparticles

- Surfactant-Directed Fabrication of Metal Oxide Nanoparticles

Surfactant-controlled synthesis introduces a key approach for producing metal oxide nanoparticles with precise control over size, distribution, and morphology. This approach utilizes the hot-injection method where a precursor solution is injected into a hot solvent containing surfactants [50]. The surfactants have multiple roles; they prevent nanoparticle agglomeration by coating them, improve colloidal stability, and selectively adsorb to crystal faces to control nanoparticle growth dynamics and the surface properties [42]. Such control has enabled the synthesis of highly uniform nanoparticles, such as iron oxides and ferrites, with advanced control over particle size down to nanometer increments, and specific shapes from spherical to cube-like structures [51]. Several key examples illustrate the versatility and effectiveness of surfactant-directed fabrication techniques in producing metal oxide nanoparticles with precise control over size, distribution, and morphology:

o Iron Oxide Nanoparticles: Achieving sizes from 6–13 nm in precise one-nanometer steps by manipulating surfactant concentrations and reaction conditions [51].
o Ferrite Nanoparticles ($MFe_{2}O_{4}$): Shape control (cube-like and polyhedron-shaped) was achieved through non-hydrolytic reactions involving metal acetylacetonates, influenced by surfactant-to-iron ratios [52].
o Complex Nanocrystal Morphologies: Examples include cone-like ZnO [45], titania nanorods, and MnO multipods [53]. $TiO_{2}$, $ZrO_{2}$, and $HfO_{2}$ exhibits the ability to create diverse nanostructures with controlled dimensions and distinctive morphologies using surfactant-mediated synthesis [46,54,55].

Despite the method’s ability to produce diverse and complex nanostructures, a significant challenge remains in the lack of universally applicable mechanistic principles that would facilitate a rational synthesis strategy. This limitation highlights the need for further exploration into organic reaction pathways and surfactant chemistry to advance the predictability and scalability of surfactant-controlled synthesis methods.

- Solvent-Directed Fabrication of Metal Oxide Nanoparticles

Solvent-controlled synthesis of metal oxide nanoparticles represents a more straightforward and highly efficient method compared to surfactant-mediated approaches, primarily due to the direct involvement of solvents in the reaction process. This method typically employs a minimal number of reactants, essentially the metal oxide precursor(s) and a common organic solvent, facilitating more straightforward characterization and elucidation of the chemical mechanisms involved. The synthesis temperatures for this method range from 50 to 200 °C, which are significantly lower than those required in the hot-injection method, contributing to improved product purity as surfactant-related impurities are absent. Given these features, the main advantages of solvent-controlled approaches include [42]:

o Improved Product Purity: The absence of surfactants eliminates issues related to nanoparticle surface accessibility, crucial for applications in catalysis and sensing, as well as reduces potential toxicity concerns associated with surface-adsorbed surfactants.
o Versatility of Metal Oxide Precursors and Solvents: The method accommodates a wide range of metal oxide precursors like metal halides, acetates, acetylacetonates, and alkoxides, as well as various solvents from oxygen-containing organic solvents (e.g., alcohols, ketones, aldehydes) to ‘inert’ solvents like toluene. This method’s flexibility permits tailored control over nanoparticle morphology and composition.
o Control Over Crystal Growth and Morphology: Organic solvents and the organic species formed during the reaction act as capping agents, controlling crystal growth and influencing particle morphology. This selectivity is crucial for achieving anisotropic crystal growth and high crystallinity.
o Halide-Free Synthesis Options: For applications where halide impurities are undesirable, solvent-controlled synthesis using non-halide precursors like metal acetates or alkoxides provides a clean alternative.

Examples of successful solvent-controlled syntheses include $SnO_{2}$ nanoparticles, which are dispersible in tetrahydrofuran and demonstrate the method’s efficiency in producing particles suitable for high-tech applications [56]. Additionally, ${BaTiO}_{3}$ nanocrystals synthesized using this approach exhibit high crystallinity, crucial for their use in piezoelectric and ferroelectric devices [57]. Indium tin oxide nanoparticles, known for adjustable dopant concentrations and excellent electrical conductivity without the need for additional annealing, further highlight the versatility and effectiveness of this synthesis method in creating conductive materials [58].

Despite the straightforward nature of the solvent-controlled synthesis, achieving phase-pure multi-metal oxides remains challenging due to the varying reactivities of different metal oxide precursors. However, the use of solvents like benzyl alcohol has demonstrated the potential to adjust precursor reactivity, enabling the synthesis of complex oxide nanoparticles with tailored properties.

### 3.2. Novel Inorganic Metal-Oxide-Based Photoresists and Synthesis Processes

Due to the higher EUV radiation absorptivity, metal-containing resist materials are becoming increasingly popular. They offer superior sensitivity, durability, and resistance to etching, alongside enhanced overall performance in resolution, pattern quality, and sensitivity. Prominent among these materials are those incorporating Hf, Zr, Zn, and Sn atoms, which significantly boost photoelectron generation when exposed to EUV radiation [18].

#### 3.2.1. Zinc-Based Inorganic Photoresists

In the approach described by Thakur et al. [19], the use of a denser zinc metallic oxo core as the inorganic component is aimed at improving the material’s EUV absorption capabilities.

The synthesis process of the photoresist involves dissolving methacrylic acid (MAA)(Figure 2a) [19] and $Zn_{4}O{\left(C_{2}HF_{3}O_{2}\right)}_{6}$, or $Zn_{4}O{\left(TFA\right)}_{6},$ where TFA is a trifluoroacetic acid, in chloroform, followed by stirring at 40 °C for 2.5 h. After evaporating the solvent, the oily residue is washed with toluene multiple times to eliminate excess MAA, resulting in a solid white compound. For the preparation of thin films, a 2% (w/v) solution of Zn(MA)(TFA) in a mixture of chloroform and propylene glycol methyl ether acetate (PGMEA) (9:1 v/v) is filtered and sonicated, then spin-coated onto substrates at specific speeds for 30 s. The films, designed for various spectroscopic analyses, are baked to remove residual solvents and their thicknesses are measured with atomic force microscopy and ellipsometry. These Zn-based oxo clusters with specific ligands show effective film formation and EUV sensitivity but experience solubility changes in thin film form due to structural adjustments [59].

To overcome the low EUV absorption of traditional organic resists, researchers have turned to metals with high photon absorption at 92 eV, like zinc. A study on a zinc-based oxo cluster [20] showed it significantly increased EUV absorption compared to conventional resists, offering a promising approach to enhance lithographic sensitivity and reduce errors.

**Figure 8:**
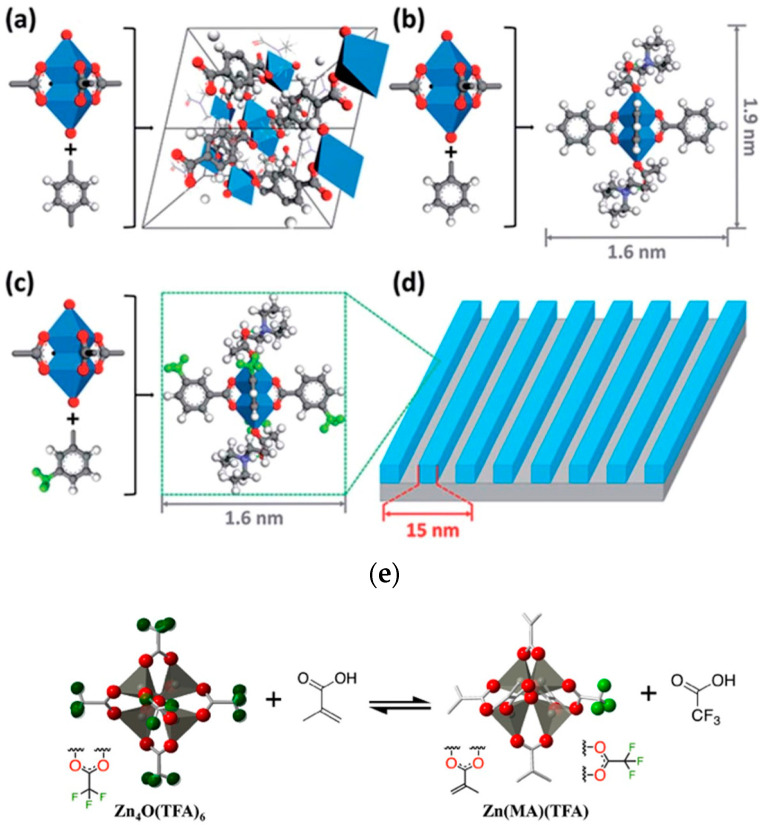
Various cluster structures synthesized using ${Zn}_{2}{\left({CO}_{2}\right)}_{4}$ as the metal unit combined with different organic ligands. (**a**) The crystal structure of MOF-2, (**b**) the structure of Zn-BA, (**c**) the structure of Zn-mTA, (**d**) 15 nm high-precision line-space (HP LS) structure can be fabricated through photoetching technology by employing Zn-mTA as a photoresist. The atomic color coding in the structures is as follows: carbon is represented in gray, oxygen in red, nitrogen in purple, hydrogen in white, and zinc in blue [60]. (**e**) The diagram of a ligand exchange reaction resulting in Zn(MA)(TFA) synthesis [61].

Thakur et al. [19] explored fluorine-rich zinc-based oxygen clusters with trifluoromethacrylate (TFMA) ligands to enhance EUV absorption, taking advantage of the significantly higher molar absorption cross-section of fluorine compared to hydrogen and carbon. Figure 8 illustrates the process of combining zinc-based oxo clusters with different organic ligands to enhance EUV absorption. This approach aims to improve lithography performance by promoting polymerization upon EUV exposure and incorporating more fluorine into the resist material.

The synthesis method of Zn(TFMA) is to dissolve Zn(TFA) and trifluoromethacrylic acid (TFMAA) in acetonitrile, stir, evaporate, and reprecipitate to remove excess acid to obtain a white solid. The solid is then dried and stored under nitrogen. The synthesis of Zn(TFMA)(MA)(TFA) involves the use of Zn(TFA)(TFMAA), TFMAA, and MAA in acetonitrile, following a similar approach to the synthesis of Zn(TFMA). Nuclear magnetic resonance (NMR) analysis details the ligand composition within the oxygen cluster. For application, the oxo cluster was formed into a thin film by spin-coating in a $CHCl_{3}:PGMEA$ solution and then baked. The silicon substrate was first treated with ozone to activate surface hydroxyl groups and then silanized using a variety of silanes, including vapor-phase applied HMDS. Liquid-phase silanization involves mixing silane in a solution of ethanol and acetic acid, soaking the silicon substrate for 40 min with stirring, and then cleaning, drying, and annealing at 130 °C for 24 h to enhance the surface properties of the deposited material [19].

In the research, two synthesis methods for zinc-based inorganic photoresists were developed to enhance the absorption of EUV radiation, aiming to improve lithography sensitivity and durability. The preparation of Zn(MA)(TFA) involves dissolution, stirring, and solvent evaporation steps, followed by film formation through spin-coating. On the other hand, the synthesis of Zn(TFMA)(MA)(TFA) introduces additional fluorine-containing compounds (TFMAA) to increase EUV absorption by incorporating more fluorine. Additionally, this method includes ozone treatment and silanization of the silicon substrate to improve film adhesion and stability. While both approaches aim to advance EUV lithography performance, the introduction of fluorine compounds adds complexity and may affect the material’s stability and polymerization rates despite potentially higher absorption efficiency.

#### 3.2.2. Tin–Oxygen Inorganic Photoresists

Tin–oxygen cage molecules can be considered as the tin equivalent of silsesquioxane compounds composed primarily of silicon [62]. Among them, hydrogen silsesquioxane HSQ is a well-known compound widely used for high-resolution patterning in electron beam and EUV lithography [63]. Tin–oxygen cage molecules were first proposed by Puff and Reuter in 1989 [64], and their use for EUV photoresist technology was explored by Cardineau et al. in 2014 [21].

**Figure 9:**
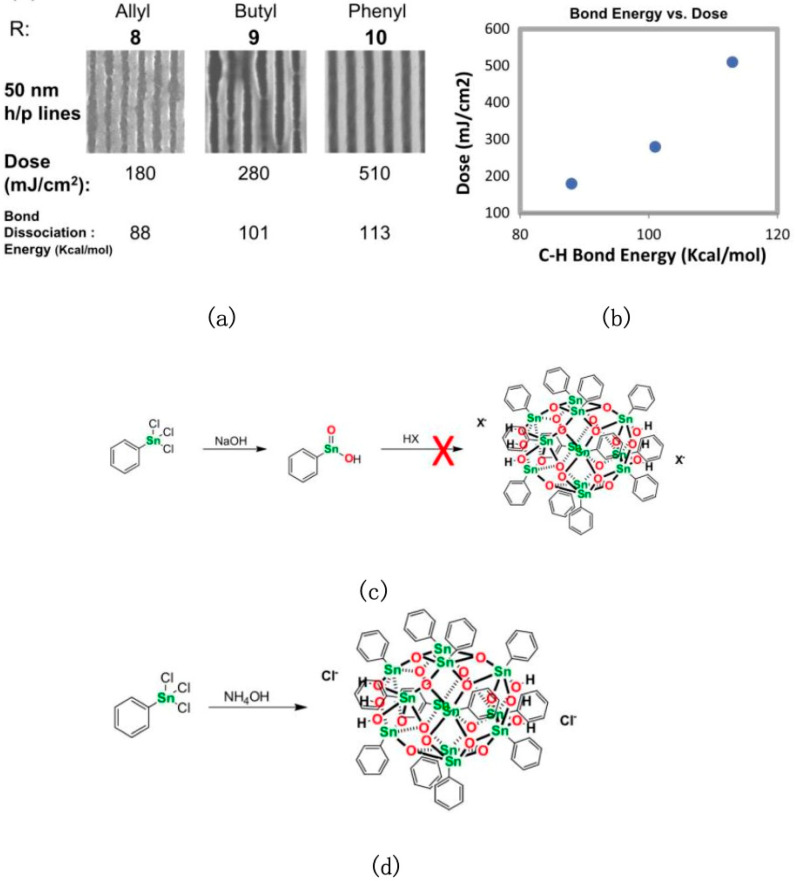
(**a**) Bond dissociation energies and photolithography results for three organic groups. (**b**) Correlation between the radical stability of organic groups and the sensitivity of clusters. (**c**) Phenyltin trichloride is converted into phenylstannic acid, and then dehydrated to synthesize tin clusters. (**d**) Phenyltin trichloride is hydrolyzed with ammonium hydroxide to form a soluble polymer material (reprinted with permission from ref. [21], 2014, Elsevier).

In the study of Cardineau et al. [21], all chemical reactions were performed under a nitrogen atmosphere. In their initial attempts to synthesize organostannoic acid and dehydrate it to form the cluster (Figure 9b), using phenyltin trichloride resulted only in an insoluble white solid, likely tin(IV) oxide due to hydrolysis. Exploring various hydrolysis methods, they found that using ammonium hydroxide with organotin trichloride was the most successful (Figure 9c). The resist formulation was created by dissolving the solid material in 2-butanone, filtered through a 0.2 µm polytetrafluoroethylene polymer (PTFE) filter [65]. These solutions were spin-coated onto 4-inch silicon wafers pre-coated with a cross-linked hydroxyethyl methacrylate/methyl methacrylate copolymer adhesive film. Cardineau et al. created a 40 nm thick resist film by fine-tuning the spin speed and formulation concentration. The films were then soft baked at 90 °C, exposed to 13.5 nm radiation, and developed using an isopropanol/water solution. Homolytic cleavage of the tin–carbon bond could occur during exposure, and Figure 9a shows the C-H bond dissociation energies for three hydrocarbons.

Jarich et al.’s study examines UV and vacuum UV fragmentation and ionization pathways of tin–oxygen cage cations and monocationic complexes to understand fundamental photochemistry that can inform the development of improved EUV photoresist materials [66]. It reveals that the counterion type significantly affects photoreactivity, suggesting that modifying the counterions and organic groups attached to the tin–oxygen cage can optimize photoresist performance.

According to a study [66], the reactions of tin–oxygen cage materials under UV and vacuum ultraviolet (VUV) light, including homolytic cleavage of tin–carbon bonds and photoionization processes, are similar to those triggered by high-energy electrons in EUV lithography. These gas-phase experiments provide critical data and theoretical insights for optimizing and improving the performance of EUV photoresist materials.

#### 3.2.3. IVB Group Inorganic-Based Photoresists

**Figure 10:**
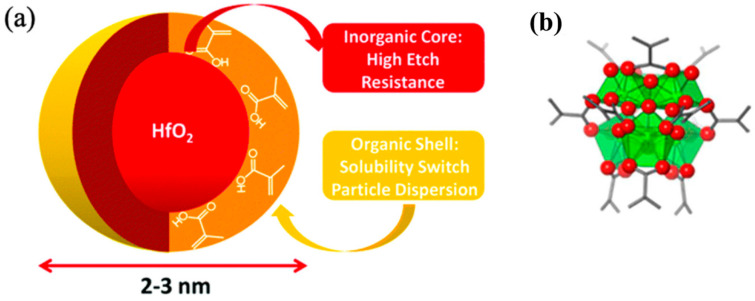
(**a**) Diagram illustrating a hybrid inorganic/organic nanoparticle featuring a $HfO_{2}$ core surrounded by an organic ligand shell (reprinted with permission from ref. [68], 1972, Royal Society of Chemistry). (**b**) Structural representation of ZrMc metal oxo clusters used as photoresists, showing the coordination geometry with polygons, oxygen atoms as red spheres, and C-C bonds as gray bars. (Reprinted with permission from ref. [8], 2002, SPIE).

**Figure 11:**
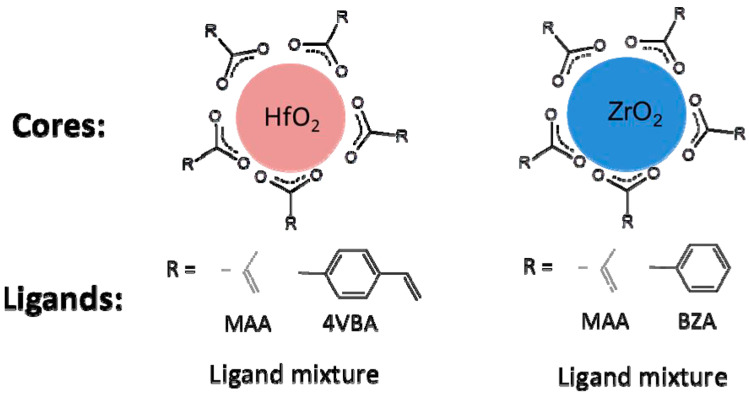
Architecture of $HFO_{2}$ and $ZrO_{2}$ nanoparticle-based photoresists (reprinted with permission from ref. [69], 2014, SPIE).

The foundational components for the inorganic $HfO_{2}$ and $ZrO_{2}$ photoresists consist of a hafnium oxide or zirconium oxide core surrounded by organic ligands (Figure 10a and Figure 11). The metal (hafnium or zirconium) oxide nanoparticles are stabilized using a range of carboxylic acids. To ensure these nanoparticles can form consistent suspensions in aqueous or organic media, two distinct synthesis techniques were employed for hafnium oxide nanoparticles:

Carboxylic-acid-ligand-stabilized hafnium oxide nanoparticles are synthesized via a methodical hydrolysis process. The procedure entailed dissolving hafnium isopropoxide in a surplus of carboxylic acid at a temperature of 65 °C, which is then followed by a gradual incorporation of a mixture of water and carboxylic acid. Upon completion of a 21 h stirring period, the addition of water facilitated the precipitation of the product. This precipitate is then subjected to centrifugation at a force of 8000× g for a duration of 5 min and subsequently is dissolved in acetone and reprecipitated with water twice. After the washing step, the product is dried at 60 °C in a vacuum to yield a white powder. To formulate the photoresist solutions, appropriate quantities of the obtained nanoparticle powder are incorporated into PGMEA, followed by adding photoactive compound and other potential additives. This mixture is then homogenized by stirring and ultrasonication to ensure uniformity. To ensure clarity and purity of the solution, any particulate matter is eliminated through filtration with a 0.2-micrometer filter membrane [25,67].

An alternative methodology that involves altering the surface characteristics of the nanoparticles is employed to derive nanoparticles with varied attributes. This is accomplished by substituting the surface-bound ligands with alternative carboxylic acids through a straightforward ligand exchange process. In a standard synthesis procedure, 2.0 g of nanoparticles are first dispersed in PGMEA. A second carboxylic acid ligand is then introduced into a small scintillation vial containing this dispersion. After this addition, the mixture is heated to 130 °C in a stirring oil bath until a transparent solution is achieved. Following this, water is added to the solution to induce the precipitation of the product, which is then subjected to several washes with acetone to eliminate any unbound acid. For the formulation of the photoresist solutions, accurate quantities of the obtained precipitate are mixed into PGMEA. This mixture is then homogenized by stirring and ultrasonication to ensure uniformity. To purge the solution of any accumulated particulates or conglomerates, it is filtered through a membrane with 0.2–micrometer pores [67].

Then, thin films are prepared for lithographic testing by applying the photoresist mixture to unprimed silicon wafers using a spin-coating technique, followed by a baking step to evaporate any residual solvent.

- The same approach is used to prepare zirconium oxide nanoparticles starting from zirconium isopropoxide as a precursor instead of hafnium isopropoxide [67].

The produced thin films can act as either positive- or negative-tone photoresists, depending on the organic ligands used on the surface of the nanoparticles during the sol–gel processing, controlling the surface chemistry of the thin film [25].

By employing a hafnium oxide system combined with methacrylic acid as the organic ligand (HfMAA), high-resolution negative-tone patterns can be achieved through the incorporation of either a photoradical initiator or a PAG as a photoactive compound. Additionally, when an extra post-exposure bake (PEB) step is introduced along with the use of an aqueous solution of tetramethylammonium hydroxide (TMAH) as the developer, the same films can be patterned in positive tone. Moreover, both positive- and negative-tone patterns can be obtained from zirconium methacrylate (ZrMAA) films by following identical procedures [67]. By manipulating the ratio of organic to inorganic content, film density, etch resistance, and absorbance can be tailored accordingly as well.

Among the category of transition metal oxides, including titanium (Ti), zirconium (Zr), hafnium (Hf), cobalt (Co), tin (Sn), and zinc (Zn), zirconium oxides ($ZrO_{2}$) emerge as the most viable alternatives for resist materials owing to their relatively low toxicity and cost [70,71]. However, Zr metal exhibits inferior absorption of EUV light compared to other transition metal oxides [8], raising doubts about the suitability of $ZrO_{2}$ as EUV photoresists. Nevertheless, when $ZrO_{2}$ is coordinated with organic ligands, its EUV absorptivity approximates that of organic photoresists [18].

Initially investigated as photoresists, $ZrO_{2}$ nanoparticles coordinated to carboxylate ligands, such as methacrylate [25], encountered challenges due to nanoparticle size, leading to scum formation and limited resolution. Subsequently, $Zr_{6}O_{4}{\left(OH\right)}_{4}{\left(methacrylate\right)}_{12}$ clusters were employed for higher-resolution lithography [72]. These clusters, approximately 1.7 nm in size, exhibit suitable characteristics for photoresist applications. $Zr_{6}O_{4}$ cluster films can be patterned with or without PAG at EUV doses ranging from 50 to 100 $mJ/cm^{2}$. Despite offering enhanced resolution, the lower sensitivity compared to $ZrO_{2}$ nanoparticles [73], in addition to the low solubility of $Zr_{6}O_{4}$ in development solvents [74], poses a significant challenge for its use as a photoresist. Various strategies, including using different ligands [74] and multiple ligands [75], have been explored to address these challenges.

Recently, a zirconium oxo cluster (Figure 10b) with methacrylate and acetate ligands was synthesized from diacetoxyzirconium (IV) oxide aqueous solution for EUV photoresist applications. This Zr oxo cluster, featuring a Zr core surrounded by methacrylate and acetate ligands, exhibits a size comparable to $Zr_{6}O_{4}{\left(OH\right)}_{4}{\left(methacrylate\right)}_{12}$ clusters but demonstrates improved solubility in a variety of organic solvents [76].

To synthesize the Zr oxo cluster, 2.0 mL of methacrylic acid (MAA) is introduced to 1.0 mL of zirconium acetate dihydrate $\left(ZrO{\left(OAc\right)}_{2}\right)$ aqueous solution under stirring, inducing precipitation. The formed precipitate is filtered using No.5A filter paper and subsequently dried under vacuum overnight [76].

For the preparation of $Zr_{6}O_{4}$ clusters, a solution comprising 2.0 g of methacrylic acid (MAA) was combined with 1.73 g of zirconium butoxide solution (80% in n-butanol). The resulting mixture was stirred overnight. The ensuing precipitates are isolated via filtration and subjected to vacuum drying overnight [76,77]. The acquired Zr oxo cluster demonstrated both high sensitivity and high resolution as a negative-tone photoresist.

## 4. Characterization Techniques for Metal-Oxide-Based Photoresists

### 4.1. Spectroscopic Analysis

Spectroscopy is most widely used technique for characterizing the composition and optical properties of EUV photoresists [78]. It provides detailed information about the material’s chemical structure, elemental composition, and interaction with light. The data collected from the spectrometer play a crucial role in evaluating and optimizing the performance of photoresists in lithographic processes [79].

For the metal-oxide-based photoresist development process, it is crucial to determine precisely the chemical reactions occurring upon its exposure to the EUV light, which influence its sensitivity. The most common tool used for this purpose is XPS, which provides a detailed elemental composition and chemical state of the elements within the photoresist. XPS measures the kinetic energy of electrons emitted from the surface of a material when irradiated with X-rays. The binding energy of these electrons provides information about the elements present and their chemical states [79]. In addition to XPS, it is effective to use X-ray absorption spectroscopy (XAS), which collects data on the total electron yield (TEY) as a function of photon energy. This information is helpful when determining the oxidation states of metal atoms and interatomic distances in the photoresist, which are crucial for predicting the behavior of the photoresist during exposure and development [79]. Diulus et al. have utilized, in their experiments on determining the chemical mechanisms behind tin-oxide-based photoresists, an instrumentation system consisting of a vacuum chamber that is coupled to an ambient-pressure gas cell by a differentially pumped electron spectrometer (VG Scienta SES-100) [79].

Another spectroscopy technique that can be used to characterize metal-oxide-based photoresists is infrared nanospectroscopy (nano-IR). It is a non-destructive method which allows quantitative and qualitative elemental composition with minimal to no damage to the photoresist film, which is the biggest advantage of this technology. However, the diffraction of the incident IR light’s long wavelength limits the spatial resolution of typical nano-IR instruments. Therefore, nano-IR is usually combined with atomic force microscopy (AFM) by integrating the IR beam onto the AFM probe [78]. As a result, the spatial resolution reaches the range of nm, making nano-IR an efficient technique for obtaining chemical data on photoresist films.

### 4.2. Microscopic Analysis

Microscopic methods are widely used for assessing pattern fidelity, LER, and other critical parameters in photolithography processes [80]. Specifically, microscopy plays a major role in characterizing metal-oxide-based photoresists, since it is especially important to assess the behavior of the photoresist and properties of the materials that it contains to reach the highest possible efficiency and accuracy in the processes of semiconductor manufacturing.

Scanning electron microscopy (SEM) is the primary imaging tool for evaluating photoresist patterns. This method provides high-resolution data at the nanometer scale, which makes it very efficient for visualizing the fine details of metal oxide photoresist patterns. It can be used at different phases of the pattern transfer process to investigate the quality and accuracy of the patterned features, such as uniformity and smoothness of the photoresist layer [81]. SEM can also be used to obtain cross-sectional images of the photoresist, providing timely details on its vertical structure, thereby accelerating the iterative process of photoresist development and increasing throughput [82].

A specific type of SEM, critical dimension scanning electron microscopy (CD-SEM), is currently the standard tool in the semiconductor industry for precise measurement of LER and LWR [83]. CD-SEM helps to control the lithography process and allows for making adjustments in the process parameters in case any deviations are detected [84]. Moreover, it provides consistent measurement repeatability at specific points across the device [85].

Other microscopic methods that are commonly used for evaluation of metal-oxide-based photoresists include transmission electron microscopy (TEM) and atomic force microscopy (AFM). The latter technique is ideal for imaging the surface topography of materials. It is often used for post-exposure analysis to understand chemical changes and sensitivity trends in a metal oxide photoresist [86].

## 5. Performance Evaluation of Metal-Oxide-Based Photoresists

### 5.1. LWR Results and Spectroscopic Analyses of Zinc-Based Photoresist

**Figure 12:**
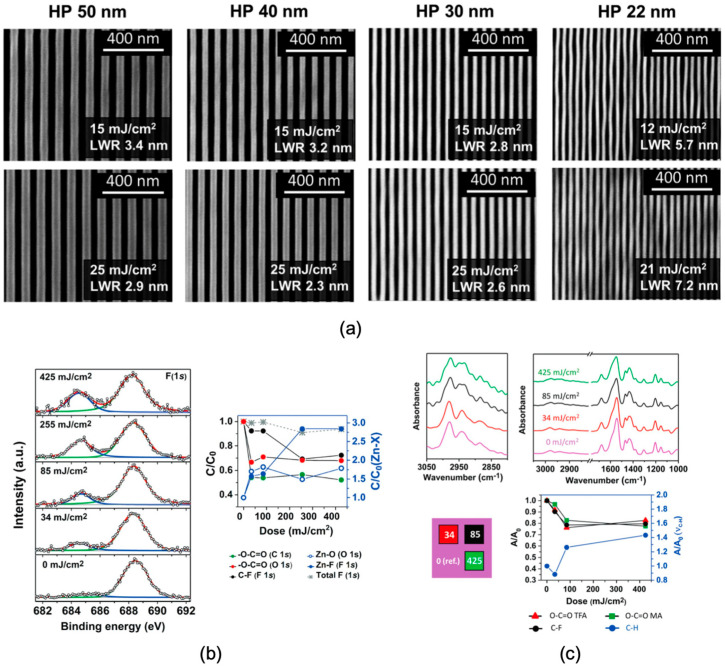
(**a**) LWR results were determined using power spectral density (PSD) analysis of SEM images (taken at 2 kV) of the line and space features. (**b**) The experimental data were fitted to F 1s high-resolution spectra for both unexposed and EUV-exposed Zn(MA)(TFA) resists, revealing variations in elemental ratios of specific components relative to Zn based on EUV dose. (**c**) FTIR spectra of Zn(MA)(TFA) resist films were analyzed before and after EUV exposure, including a schematic of the exposed sample showing regions subjected to different EUV doses and the changes in peak areas related to MA and TFA ligands as a function of dose compared to the unexposed region [59].

A study by Thakur et al. [59] investigated fluorine-rich zinc-based oxygen clusters with TEFMA ligands to improve EUV absorption and provided a thorough evaluation of the Zn(MA)(TFA) photoresist’s performance for EUV lithography. The $Zn\left(MA\right)\left(TFA\right)$ resist effectively created dense line/space (L/S) patterns with half-pitches (HPs) ranging from 22 to 50 nm, requiring a low exposure dose of around 20 $mJ/cm^{2}$, which meets current EUV resist standards. The pattern transfer was clean, with no scumming observed between the lines. The aspect ratios were 1:3 for 50 nm, 30 nm, and 22 nm HPs, and about 1:2 for 40 nm HP (Figure 12a). Most HP values met the industry requirement of LWR being less than 20% of the CD [87]. Achieving low LWR while using high-sensitivity resists (where high sensitivity equates to low exposure dose) is a significant challenge for EUV resists due to the effects of photon shot noise [88].

Spectroscopic analyses revealed the chemical changes in the Zn(MA)(TFA) photoresist upon EUV exposure and demonstrated its exceptional performance. FTIR spectroscopy showed significant reductions in the carboxylate group peaks of TFA and MA ligands, particularly at 1676 cm^−1^ and 1544 cm^−1^, after EUV exposure. Additionally, the C-F stretching vibration peaks (1205 cm^−1^ and 1155 cm^−1^) decreased, while the aliphatic C-H stretching peaks (2929 cm^−1^ and 2885 cm^−1^) increased, indicating decarboxylation of the TFA and MA ligands and C-F bond cleavage [15]. UV–Vis absorption spectroscopy further confirmed these changes, with the absorption band at 198 nm in the unexposed material bleaching as the EUV dose increased (Figure 12b,c), reflecting the loss of double bonds in the MA ligands and the formation of cross-linked saturated carbon chains. XPS analysis revealed significant changes in the high-resolution spectra of C 1s, O 1s, and F 1s, including the formation of Zn-F bonds and a reduction in carboxylate (O-CQO) components.

These spectroscopic results indicate that the Zn(MA)(TFA) photoresist possesses high sensitivity, low dose requirements, high resolution, and excellent roughness control. The significant chemical reactions occurring at low EUV doses demonstrate its high sensitivity and low dose needs. The cross-linking of MA ligands forming saturated carbon chains confirms its high-resolution capabilities. Additionally, the formation of Zn-F bonds and other cross-linking reactions enhance the edge clarity and consistency of the patterns, demonstrating excellent roughness control. These properties make the Zn(MA)(TFA) photoresist a highly promising candidate for EUV lithography.

### 5.2. Topographical and Chemical Analysis of Tin–Oxygen Inorganic Photoresists

The dual-tone property of the tin oxo cage $\left[{\left(BuSn\right)}_{12}O_{14}{\left(OH\right)}_{6}\right]{\left(OH\right)}_{2}$ photoresist was reported by Zhang et al. [22]. The TinOH photoresist was applied to pre-treated silicon wafers via spin-coating, creating a thin layer around 20 nm thick. This was followed by a pre-bake to eliminate residual solvents. The samples were then subjected to various doses of EUV or electron beam radiation. After exposure, the samples underwent a post-exposure bake at 150 °C for 2 min, were developed in a mixture of isopropanol and water (2:1) for 30 s, rinsed for 30 s, and finally hard baked at 150 °C for 1 min to ensure all solvents were removed.

**Figure 13:**
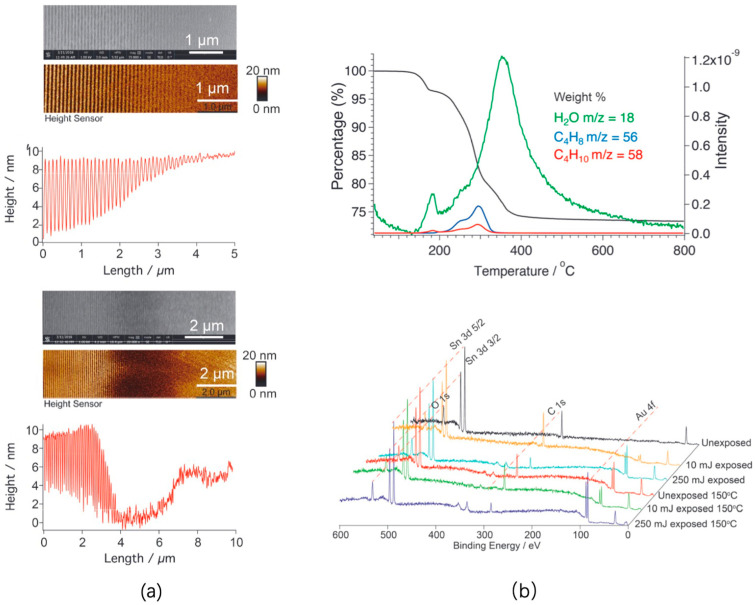
(**a**) SEM and AFM images of tin oxo cage thin films patterned with 3.6 mJ/cm^2^ and 53.5 mJ/cm^2^ EUV doses, along with their corresponding cross-sectional profiles. (**b**) The spectra of TinOH thin layers with various EUV exposures and post-exposure baking, TGA of TinOH powder, highlighting weight changes and the loss of water, butene, and butane with temperature [89].

Topographical analysis using AFM and SEM revealed that low doses of electron beam exposure (e.g., 50 µC/cm^2^) resulted in positive-tone patterns, with the exposed areas being completely removed. As the exposure dose increased (e.g., 400 µC/cm^2^ and 1000 µC/cm^2^), negative-tone patterns emerged, with the unexposed areas showing reduced thickness, forming clear negative-tone patterns (Figure 13a). Similar dual-tone behavior was observed with EUV exposure: low doses (e.g., 3.6 mJ/cm^2^) produced positive-tone patterns, while higher doses (e.g., 53.5 mJ/cm^2^) resulted in negative-tone patterns.

Chemical analysis using XPS showed significant carbon loss at high doses due to Sn-C bond cleavage, which explains the formation of negative-tone patterns. TGA results (Figure 13b) indicated that water loss during post-exposure baking at 150 °C reduced the solubility of the unexposed film, aiding the transition from positive to negative tone.

The evaluation highlights that TinOH photoresist exhibits dual-tone properties under varying doses of EUV and electron beam exposure, enabling complex structure formation in a single photoresist layer. This dual-tone capability enhances patterning flexibility and precision [90]. Additionally, TinOH shows high sensitivity to EUV and electron beam exposure, allowing clear pattern formation at low doses, crucial for high-resolution lithography. TGA and XPS analyses also indicate some thermal stability but chemical changes at high doses, such as carbon loss and material densification, affecting solubility and development. Overall, TinOH demonstrates excellent performance, including dual-tone properties, high sensitivity, and high resolution, making it a promising candidate for next-generation high-precision lithography.

### 5.3. Analysis of IV B Group Inorganic-Based Photoresists

Markos’s study showed that Zr-based hybrid resists can achieve 30 nm resolution patterning with exceptionally high sensitivity [24]. The process began with the synthesis and preparation of the nanoparticles, where ${HfO}_{2}$ and $ZrO_{2}$ nanoparticles were synthesized through a controlled chemical process to ensure uniform sizes and proper dispersion characteristics. After exposure and development, the developed patterns were analyzed using SEM, focusing on parameters such as resolution, LER, and overall pattern fidelity, providing detailed insights into the quality of the patterns.

The nanoparticle photoresists demonstrated high-resolution capabilities, producing patterns with line widths as small as 22 nm, essential for advanced semiconductor manufacturing. The patterns exhibited low LER, with roughness values below 3 nm, critical for ensuring the accuracy and reliability of semiconductor components. The photoresists showed high sensitivity to EUV radiation, achieving effective patterning at the specified dose of 25 mJ/cm^2^, which helps in reducing exposure time and increasing production efficiency. Additionally, the nanoparticle photoresists maintained their integrity during the etching process, showing excellent resistance to etching chemicals, crucial for preserving pattern fidelity during semiconductor fabrication. The spin-coating process resulted in highly uniform and consistent coatings on the wafers, ensuring reliable and repeatable patterning results across multiple wafers.

The inorganic photoresists composed of $HfO_{2}$ and $ZrO_{2}$ nanoparticles reported by Markos et al. exhibit exceptional etch resistance, surpassing polymer resists by up to 25 times, and therefore enabling the processing of extremely thin films (<40 nm). As a result, resolution limits below 20 nm can be achieved without encountering pattern collapse. Moreover, the nanoparticles’ small size (<5 nm) contributes to minimal LER and low absorbance at EUV wavelengths. Achieving high-resolution patterning (<30 nm) with remarkable sensitivity and low LER underscores the efficacy of these inorganic resists, particularly in EUV lithography applications [25,67].

Further investigations reveal the superior etch resistance of nanoparticle films, attributed to their thermal and chemical stability. Additionally, the hybrid composition of these films allows for precise control over absorbance, optimizing lithographic performance. The successful patterning of ZrMAA films with negative tone, utilizing a record-low EUV dose of 4.2 mJ/cm², further highlights the remarkable sensitivity of these materials in EUV lithography. Moreover, beyond enhanced etch resistance, these inorganic photoresists offer advantages such as improved depth of focus (DOF) and reduced LER compared to polymer alternatives. Additionally, the successful pattern transfer into Si substrates using $SF_{6}/O_{2}$ etching underscores the efficacy of these inorganic photoresists in practical applications, further establishing their superiority over polymer counterparts [25,67].

In Kataoka et al.’s study about zirconium-oxo-cluster-based EUV photoresists, thermogravimetric analysis (TGA) conducted in air aimed to further characterize Zr oxo and $Zr_{6}O_{4}$ clusters. Both cluster types exhibited stepwise mass loss between 100 °C and 220 °C, followed by thermal decomposition above 300 °C. At 220 °C, the Zr oxo cluster demonstrated a mass loss of 47.1%, slightly less than the $Zr_{6}O_{4}$ cluster’s 57.2%, suggesting a smaller ligand fraction in the former. Analysis of powder X-ray diffraction (PXRD) patterns revealed well-defined molecular crystals for $Zr_{6}O_{4}$ clusters, contrasting with the lower crystallinity observed in Zr oxo clusters. Matrix-assisted laser desorption/ionization time-of-flight mass spectrometry (MALDI-TOFMS) measurements showed similar mass spectra for both clusters despite differences in TGA and PXRD results. Dynamic light scattering (DLS) measurements in methanol solution indicated comparable sizes for Zr oxo (2.0 nm) and $Zr_{6}O_{4}$ clusters (1.8 nm), with a slightly broader size distribution for the former. Notably, the $Zr_{6}O_{4}$ cluster size corroborated previous reports, while the smaller size of Zr oxo clusters (compared to reported Zr nanoparticles) suggests suitability for high-resolution patterning in photoresist applications [76].

The solubility of photoresists was evaluated across various solvents (n-hexane, toluene, chloroform, PGMEA, butyl acetate, ethanol, and methanol). With the exception of n-hexane, all solvents formed transparent solutions with the Zr oxo cluster, which exhibited rapid dissolution in many instances. Conversely, the $Zr_{6}O_{4}$ cluster demonstrated lower solubility in some of the selected solvents, requiring more dissolving time. Presumably, the enhanced solubility of the Zr oxo cluster can be attributed to its lack of crystallinity. In PGMEA, a common coating solution, the solubility of the Zr oxo cluster exceeded 20 wt%, while that of the $Zr_{6}O_{4}$ cluster remained below 1.4 wt%. Similarly, in butyl acetate, a prevalent development solution for EUV resists, the solubility of the Zr oxo cluster surpassed 20 wt%, whereas that of the $Zr_{6}O_{4}$ cluster was below 1.5 wt%. Thus, the markedly higher solubility of the Zr oxo cluster across a broad spectrum of solvents renders it highly suitable for application as a photoresist [76].

To evaluate the efficacy of the Zr oxo cluster as a photoresist, an open-frame exposure test was conducted, yielding normalized contrast curves characteristic of negative-tone photoresists. The Zr oxo cluster film showed notably higher sensitivity compared to $Zr_{6}O_{4}$ cluster film, with a threshold dose of approximately $3\;mJ/cm^{2}$ for the Zr oxo cluster compared to approximately $5\;mJ/cm^{2}$ for the $Zr_{6}O_{4}$ cluster. Additionally, the minimum dose at full height for the Zr oxo cluster stood at approximately $13\;mJ/cm^{2}$, while for the $Zr_{6}O_{4}$ cluster, it was approximately $25\;mJ/cm^{2}$. While the sensitivity of the $Zr_{6}O_{4}$ cluster aligns with prior reports, that of the Zr oxo cluster demonstrates comparable or superior performance relative to other reported photoresists [76].

**Figure 14:**
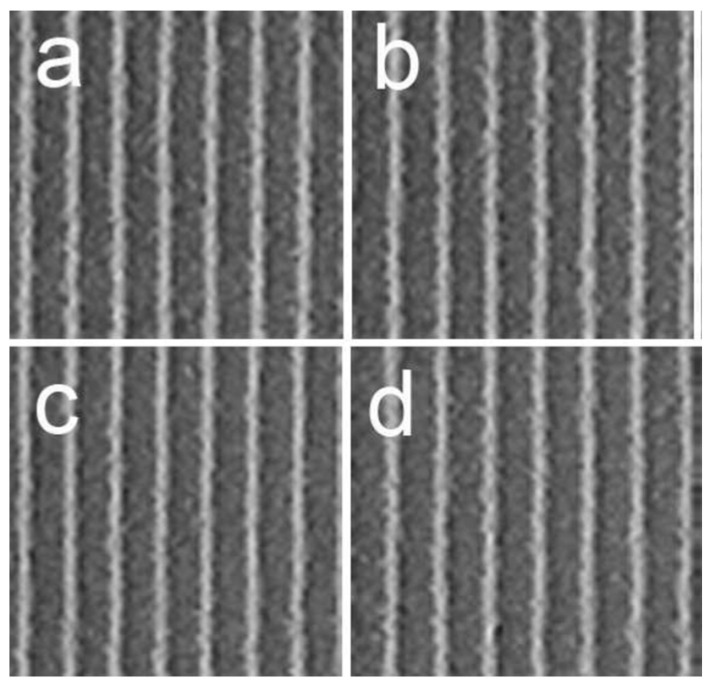
SEM images showing patterns of Zr clusters with varying line spacings (1:5 ratio): (**a**) Lines composed of Zr oxo clusters with a 15 nm pattern width; (**b**) Lines of Zr oxo clusters with a 16 nm pattern width; (**c**) Lines formed from $Zr_{6}O_{4}$ clusters, each 15 nm wide; (**d**) $Zr_{6}O_{4}$ cluster lines, each 16 nm in width (reprinted with permission from ref. [76], 2022, John Wiley and Sons).

Further investigation into Zr clusters of 15 nm and 16 nm widths at pitches of 1:5 revealed noteworthy resolution capabilities (Figure 14). The Zr oxo cluster resolved lines of 15 nm width at a dose of $22\;mJ/cm^{2}$, while the $Zr_{6}O_{4}$ cluster achieved the same with a dose of $46\;mJ/cm^{2}$. SEM imaging indicated satisfactory line width roughness for both clusters. Notably, the superior sensitivity of the Zr oxo cluster, as indicated by the contrast curve, was accompanied by the absence of observable scum or defects, a notable distinction from other nanoparticle photoresists. It is pertinent to mention that the films underwent exposure to EUV light in the absence of PAG. The Zr oxo cluster exhibits sustained line width roughness when patterned at doses sufficiently low for industrial applications ($22\;mJ/cm^{2}$) [76].

## 6. Applications and Future Prospects

### 6.1. Zinc-Based Inorganic Photoresist in Nanofabrication and 3D Printing

#### 6.1.1. Zinc-Based Photoresist for High-Resolution EUVL and Nanofabrication

Based on previous work [24] on Zr- and Hf-based photoresists, Xu et al. [60] from Ober’s group have developed Zn-based nanoparticles, achieving sub-15 nm patterns via EUV lithography. These zinc-based photoresists, akin to their $ZrO_{2}$ and $HfO_{2}$ counterparts, feature a ZnO core with an organic shell of acid and base ligands, offering fine particle size, solubility in spin-coating solvents, superior film formation, and effective patterning under UV and e-beam exposure. The zinc-based clusters facilitate the creation of nanostructures smaller than 15 nm, advancing the limits of nanofabrication to the scale of the clusters themselves. This advancement aids contemporary microfabrication methods by enabling the production of more refined structures compared to those achievable with traditional polymer-based materials [91].

#### 6.1.2. Zinc-Based Photoresist for 3D Printing

These photoresists share similarities with toluic-acid-based alternatives, featuring small particle sizes, excellent solubility for spin-coating, and effective film formation, with decent patterning under deep-UV light. Unlike metal oxide nanoparticles using a photoacid generator (PAG) mechanism, these show enhanced sensitivity and resolution with a photoradical generator. Their strong EUV absorption and the radical mechanism under EUV suggest their potential for 3D printing, achieving notable results like 50 μm resolution and fast printing [91].

**Figure 15:**
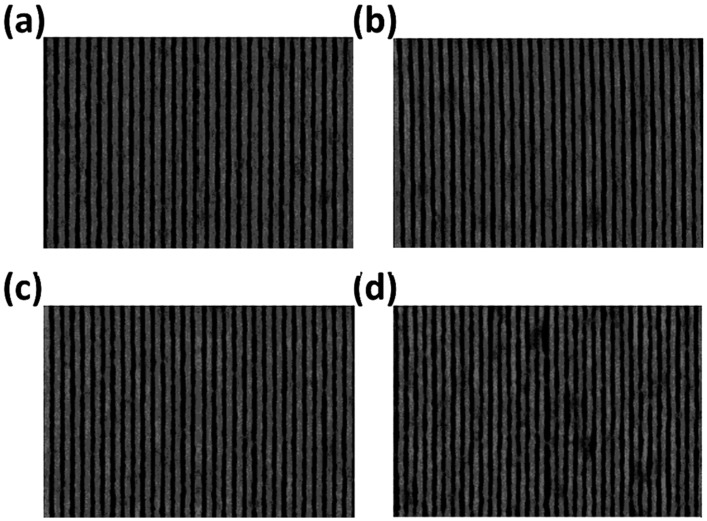
SEM images of Zn-mTA cluster line and space dense patterns fabricated via EUV exposure: (**a**) 16 nm at a dose of 45 mJ/cm^2^, (**b**) 15 nm at a dose of 47 mJ/cm^2^, (**c**) 14 nm at a dose of 36 mJ/cm^2^, and (**d**) 13 nm at a dose of 35 mJ/cm^2^ [60].

This potential is described by the SEM images depicting good line and space patterns having a 13, 14, 15, and 16 nm feature sizes with a dose of 35, 36, 47, and 45 $mJ\;cm^{-2}$, respectively. Figure 15 shows the SEM images of aforementioned feature sizes with specific EUV doses.

In conclusion, these photoresists exhibit exceptional potential for advanced lithography and 3D printing, thanks to their superior sensitivity, resolution, and strong EUV absorption. SEM images confirm precise patterning with feature sizes down to 13 nm, demonstrating their capability in nanofabrication.

### 6.2. Tin–Oxygen Cluster Inorganic Photoresist for High-Resolution Lithography

**Figure 16:**
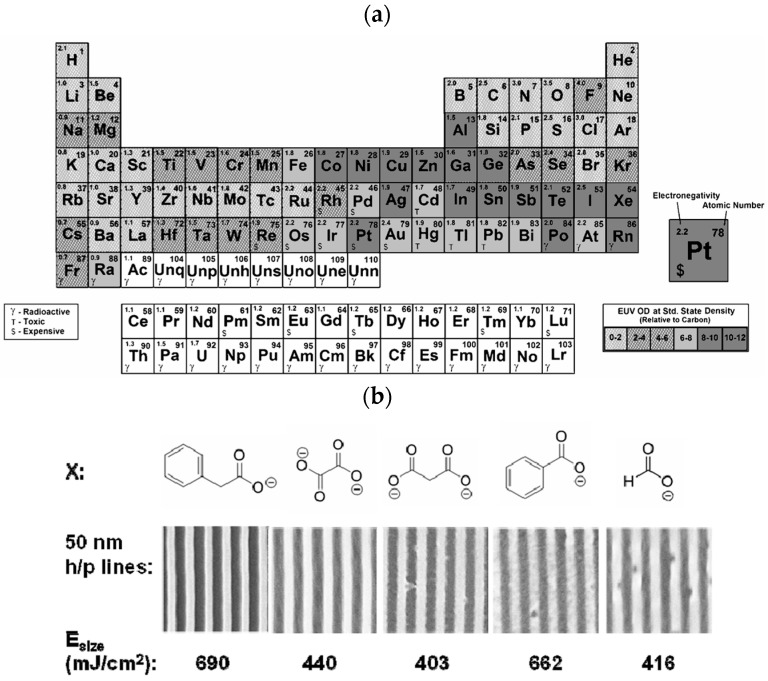
(**a**) Optical density (EUV) mentioned in periodic table. (**b**) Tin clusters were examined using lithographic techniques to determine their relative sensitivity (reprinted with permission from ref. [92], 2014, SPIE).

Tin–oxygen cluster inorganic photoresists have shown promising applications in EUV lithography. Since tin and oxygen have higher absorption densities than carbon, these materials have the ability to absorb EUV photons efficiently (refer to Figure 16a). The increased optical densities of these tin oxo clusters enable resists to more effectively harness EUV photons during EUV lithography. This enhanced efficiency results in superior lithographic performance, characterized by improved sensitivity and reduced shot noise. This property makes them suitable for high-resolution lithography applications. The photoreaction mechanism in these clusters involves activation and aggregation of organic ligands on the surface of tin clusters under extreme ultraviolet exposure, exhibiting typical properties of negative photoresists [92].

These clusters have been subjected to various experimental setups to understand their fragmentation and ionization pathways under UV and vacuum UV light. Studies have shown that depending on the type of counteranion attached to the cluster, the photochemical behavior can differ significantly, affecting the sensitivity and resolution of the photoresist. Changing these anions through anion exchange reactions can alter photoresist properties, demonstrating a tunable approach to optimizing photolithographic performance. A study indicated that the reactivity of the clusters was more closely related to the strength of the tin–carbon bonds. Specifically, the cluster with the weakest tin–allyl bond exhibited the highest sensitivity. This suggests that homolytic cleavage of the tin–carbon bond could generate tin-centered radicals, which might react with adjacent clusters, creating cross-links and causing cluster agglomeration. Furthermore, larger counterions would increase the spacing between clusters, thereby hindering tin–tin bond formation and decreasing the resist sensitivity [93] (Figure 16b).

In summary, tin–oxygen cluster inorganic photoresists exhibit excellent potential for EUV lithography due to their high EUV photon absorption, improved sensitivity, and reduced shot noise. Their tunable photochemical properties, influenced by the type of counteranion, offer a pathway for optimizing lithographic performance. This makes them highly suitable for high-resolution applications, as demonstrated by their effective patterning capabilities and reactivity under extreme UV exposure.

### 6.3. IVB Group Inorganic-Based Photoresist

IVB group metal oxo clusters (MOCs) are essential in EUV lithography due to their unique properties and heightened sensitivity to EUV photons, enabling precise patterning in semiconductor devices. These clusters enhance EUV photon absorption, making them effective photoresists in photolithography for creating fine features on semiconductor wafers crucial for advanced microelectronics. Beyond photolithography, MOCs are utilized in optical devices like waveguides and photonic circuits, nanoscale electronic components, and improving the performance of sensors and detectors. They also play a role in quantum dots, with applications in quantum computing and imaging [18,19].

#### 6.3.1. Zirconium Hafnium Oxide Thin Film as CMOS-Compatible Pyroelectric Infrared Sensor

**Figure 17:**
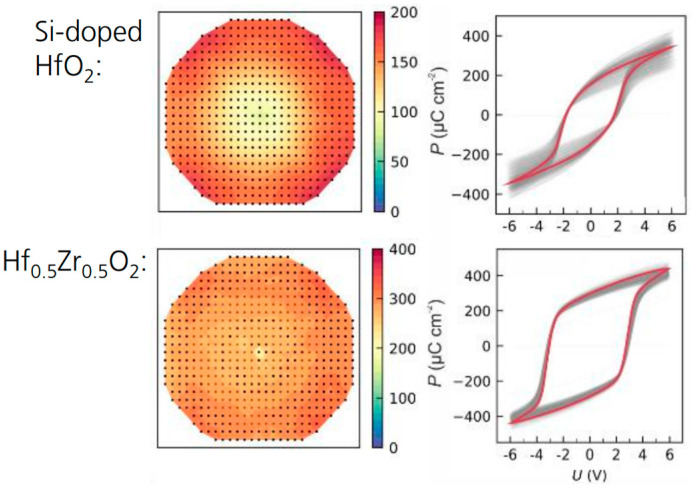
Hysteresis curve comparison of Si-doped and Hf-Zr-based sensors with better area increase on the substrate [94].

The application of hafnium zirconium oxide ($Hf_{1-x}\;Zr_{x}O_{2}$) as a pyroelectric infrared sensor, i.e., generates electric signal due to temperature change, represents an eco-friendly alternative to sensors traditionally based on lead-containing materials. In this context, incorporating complementary metal oxide semiconductor (CMOS) technology facilitates integrated sensor circuit development, providing scalability and cost efficiency. SEM demonstrates that holes with a 500 nm diameter and an 8 µm depth exhibit conformal deposition. Utilizing TiN electrodes and photolithography, capacitor structures are formed, exhibiting uniformity and a substantial remnant polarization of up to $331\;µC{cm}^{-2}$, corresponding to a 15 times greater area on the nanostructured substrate. Ferroelectric hysteresis measurements and pyroelectric analysis, involving a sinusoidal temperature oscillation, confirm the pyroelectric origin of the signal (Figure 17). The devices demonstrate significant pyroelectric coefficients of $-475\;µCm^{-2}K^{-1}$, surpassing those of lead zirconate titanate (PZT). Based on experimental findings, ($Hf_{1-x}\;Zr_{x}O_{2}$) appears as a potential material for pyroelectric applications in the future, offering both environmental sustainability and enhanced performance [94].

#### 6.3.2. Zirconium Oxide for Ultrahigh-Speed Printing

The application of zirconium oxide hybrid photoresist in ultrahigh-speed printing for precise nanoscale additive manufacturing demonstrates a significant advancement over current two-photon lithography techniques. While traditional methods can achieve nanoscale resolution, they are often too slow for large-scale practical use. This approach utilizes a highly sensitive zirconium oxide hybrid-(2,4-bis(trichloromethyl)-6-(4-methoxystyryl)-1,3,5-triazine) (ZrO_2_-BTMST) photoresist system, enabling a remarkable printing speed of 7.77 m/s, which is three to five orders of magnitude faster than conventional polymer-based photoresists.

By integrating a polygon laser scanner-based two-photon lithography machine with a linear stepping speed approaching 10 m/s, a 1 cm^2^ square raster was successfully fabricated in approximately 33 min. The ZrO_2_-BTMST photoresist’s small chemical components allow patterning with high precision, achieving line widths as narrow as 38 nm. Analysis indicates that this enhanced sensitivity is due to an efficient light-induced polarity change in the ZrO_2_ hybrid material. This breakthrough suggests that the exceptional sensitivity of this organic–inorganic hybrid photoresist could enable the development of viable large-scale nanofabrication technology for additive manufacturing [95].

### 6.4. Market Opportunities and Commercial Prospects

#### 6.4.1. International Market

As the industry is both capital and technology intensive, the global market for photoresists is dominated by a few large companies based in Japan, the United States, and Europe. Japanese and American companies together hold over 85% of the market share. Major international players in the photoresist market include JSR, Tokyo Ohka Kogyo (TOK), Dow Chemical, Fujifilm Electronic Materials, Shin-Etsu Chemical, Merck, Sumitomo Chemical, and Nissan Chemical, reflecting high market concentration. Data from 2019 indicate that the top five manufacturers controlled 87% of the global photoresist market. Four of these top five companies are Japanese—JSR, Tokyo Ohka Kogyo, Shin-Etsu Chemical, and Fujifilm Electronic Materials—collectively accounting for 72% of the market. Overall, Japanese companies hold more than 75% of the market share. Specifically, in the ArF, KrF, and g/i-line photoresist segments, Japanese manufacturers dominate with market shares of 93%, 80%, and 61%, respectively, underscoring their strong position in the high-end market [96,97].

#### 6.4.2. Commercial Prospect

The commercialization prospects of metal-based photoresists are promising due to their exceptional performance in high-resolution and high-precision lithography applications. However, addressing challenges related to cost and environmental impact is crucial for their widespread adoption. Companies like Inpria and JSR are at the forefront of developing these advanced materials, with ongoing research continuing to highlight their potential benefits for the semiconductor industry.

Inpria and Interuniversity Microelectronics Centre (IMEC) have achieved a significant milestone in the practical implementation and scaling of metal-based photoresists from laboratory research to full-scale semiconductor fabrication. By integrating Inpria’s directly patternable metal oxide hard mask as a robust, high-resolution photoresist into IMEC’s N7 BEOL block mask process module, they meticulously examined both lithography and etch patterning outcomes. This collaboration leveraged the high differential etch resistance of metal oxide photoresists, enabling process simplification and cost reduction. The joint effort included a detailed review of imaging results, process windows, underlayer integration, etch transfer, cross-sectional analysis, etch equipment integration to prevent cross-metal contamination, and selective resist strip processes. Additionally, they reported initial success with a higher-sensitivity Inpria resist, achieving a dose to size of 19 mJ/cm^2^ and printing pillars as small as 21 nm. This marks a crucial step in advancing EUV lithography for next-generation semiconductor manufacturing [98].

Inpria, now acquired by JSR Corporation, specializes in metal oxide photoresists designed for EUV lithography. Their products are known for their high-resolution capabilities and are being integrated into advanced semiconductor manufacturing processes. JSR is actively developing a range of lithography materials, including EUV photoresists, for next-generation semiconductor manufacturing processes targeting the 10 nm node and smaller. As major players in the photoresist industry, both Inpria and JSR continue to innovate and drive the development of metal-containing photoresists for future semiconductor processes [99].

### 6.5. Future Prospects

Future perspectives of inorganic metal-oxide-based photoresists for EUVL hold significant promise in advancing semiconductor manufacturing processes. By harnessing the unique properties of metal oxides, such as high chemical reactivity and precise tunability, these photoresists can enable the fabrication of semiconductor devices with smaller feature sizes and higher resolution. Future research should focus on optimizing ligand chemistry, including the introduction of novel ligands and combinations to enhance film density and control cross-linking during EUV exposure. The exploration of multi-metal oxo clusters offers potential for better control over solubility and EUV absorbance, which could balance sensitivity, resolution, and LER. Advanced synthesis techniques, such as the refinement of non-aqueous sol–gel processes, are crucial for improving the quality and consistency of metal oxide nanoparticles. In-depth mechanistic studies will elucidate the chemical processes during lithography, enabling the optimization of exposure and development parameters. Investigating the nature and impact of insoluble materials formed during lithography will further refine photoresist formulations. Compatibility with novel semiconductor materials, such as new substrates, interconnects, and dielectrics, can integrate inorganic photoresists into advanced device architectures. Additionally, the environmental benefits of inorganic photoresists, with lower outgassing and reduced environmental impact compared to traditional organic resists, should be explored. Beyond semiconductor manufacturing, these photoresists have potential applications in pyroelectric infrared sensors, optical devices, and 3D printing, offering high stability and resolution capabilities. By addressing these specific research directions, the field can advance significantly, driving improvements in EUV lithography and other advanced technologies.

## 7. Conclusions

Metal-oxide-based photoresists have shown significant promise in overcoming the limitations of traditional organic photoresists. This review has highlighted significant progress in the development and application of inorganic metal-oxide-based photoresists, focusing on zinc-based, tin–oxygen, and IVB group photoresists. These photoresists exhibit unique properties that enhance their chemical reactivity and precise patterning capabilities, making them suitable for EUVL. Zinc-based photoresists, utilizing ZnO as the photoactive component, show enhanced EUV absorption and mechanical stability, crucial for achieving high-resolution patterns. The incorporation of fluorine-rich ligands in these resists further improves their sensitivity and durability. Tin–oxygen photoresists, with their tin oxo clusters, demonstrate high EUV photon absorption and effective negative-tone properties, driven by the homolytic cleavage of Sn–C bonds and the formation of cross-linked structures. IVB group metal oxides, particularly ${HfO}_{2}$ and ${ZrO}_{2}$, enhance etch resistance and resolution due to their high absorptivity and stability under EUV exposure.

Advanced synthesis techniques such as ALD, ASD, and VPI have been pivotal in achieving uniform, atomically precise coatings that enhance the performance of these photoresists. The development of hybrid inorganic–organic structures, MALD, and non-aqueous sol–gel processes has further improved the sensitivity, mechanical strength, and resolution of metal-oxide-based photoresists.

Performance evaluations reveal that these photoresists possess excellent potential for high-resolution lithography. Zinc-based photoresists achieve sub-15 nm patterns, maintaining low LWR and significant chemical changes under EUV light, ensuring high resolution and roughness control. Tin–oxygen photoresists exhibit high sensitivity and reduced shot noise due to their high EUV photon absorption, with tunable properties through counteranion modification. IVB group resists, particularly those using Zr and Hf oxides, show superior etch resistance and minimal LER, capable of producing patterns with resolutions below 30 nm. The Zn(MA)(TFA) photoresist demonstrates high sensitivity and the ability to form fine line/space patterns at low exposure doses. The TinOH photoresist’s dual-tone properties and high sensitivity to EUV and electron beam exposure provide greater flexibility and precision in patterning. Zirconium-based hybrid photoresists are capable of patterning at a 30 nm resolution with low LER, maintaining integrity during the etching process.

The absorptivity of metal-containing resists significantly influences their effectiveness. Tin-based compounds exhibit absorption coefficients two to three times greater than conventional organic CARs, enhancing their sensitivity. These findings highlight the ability of metal-oxide-based photoresists to meet the stringent requirements for next-generation semiconductor devices, including high sensitivity, low dose requirements, and excellent roughness control.

The broad applicability of these photoresists extends beyond traditional photolithography. Zinc-based photoresists show potential for nanofabrication and 3D printing, offering fine particle size, excellent solubility, and strong EUV absorption for creating intricate nanostructures. Tin–oxygen clusters’ enhanced EUV absorption and tunable photochemical properties make them suitable for high-resolution applications, while IVB group metal oxo clusters demonstrate versatility in photolithography, optical devices, nanoscale electronics, and quantum computing. Hafnium zirconium oxide thin films have also been identified as eco-friendly alternatives for pyroelectric infrared sensors, integrating well with CMOS technology for scalable and cost-efficient sensor development.

Market opportunities for metal-based photoresists are promising, with major players like Inpria and JSR leading advancements in high-resolution lithography materials. Collaborative efforts, such as those between Inpria and IMEC, highlight significant progress in transitioning from research to full-scale semiconductor fabrication, achieving high-resolution patterning and cost reductions. The dominance of Japanese and American companies in the global market underscores the high commercial potential of these advanced materials.

Future research should focus on understanding the intricate chemical mechanisms and the nature of the insoluble materials formed during the EUV exposure process. Additionally, further refinement of the resolution and sensitivity of metal-oxide-based photoresists will expand their applicability in various fields, such as pyroelectric infrared sensors, optical devices, and 3D printing. Continued advancements in spectroscopic and microscopic characterization methods will be essential for the efficient development and enhancement of these photoresists. By addressing the remaining challenges and leveraging the unique properties of metal-oxide-based photoresists, EUV lithography can receive development impetus that would contribute to further advancements in the semiconductor industry.
